# System-wide vitreous proteome dissection reveals impaired sheddase activity in diabetic retinopathy

**DOI:** 10.7150/thno.72947

**Published:** 2022-09-11

**Authors:** Asfa Alli-Shaik, Beiying Qiu, Siew Li Lai, Ning Cheung, Gavin Tan, Suat Peng Neo, Alison Tan, Chiu Ming Gemmy Cheung, Wanjin Hong, Tien Yin Wong, Xiaomeng Wang, Jayantha Gunaratne

**Affiliations:** 1 Institute of Molecular and Cell Biology (IMCB), Agency for Science, Technology and Research (A*STAR), Singapore 138673.; 2 Singapore Eye Research Institute (SERI), Singapore 169856.; 3 Singapore National Eye Centre (SNEC), Singapore 168751.; 4 Centre for Vision Research, Duke-NUS Medical School, Singapore 169857.; 5 Tsinghua Medicine, Tsinghua University, Beijing China 100084.; 6 Yong Loo Lin School of Medicine, National University of Singapore, Singapore 117594.

**Keywords:** ADAM10, ectodomain shedding, diabetic retinopathy, sheddase, vitreous proteome

## Abstract

**Rationale:** Diabetic retinopathy (DR) is a major complication of diabetes mellitus causing significant vision loss. DR is a multifactorial disease involving changes in retinal microvasculature and neuronal layers, and aberrations in vascular endothelial growth factors (VEGF) and inflammatory pathways. Despite the success of anti-VEGF therapy, many DR patients do not respond well to the treatment, emphasizing the involvement of other molecular players in neuronal and vascular aberrations in DR.

**Methods:** We employed advanced mass spectrometry-based proteome profiling to obtain a global snapshot of altered protein abundances in vitreous humor from patients with proliferative DR (PDR) in comparison to individuals with epiretinal membrane without active DR or other retinal vascular complications. Global proteome correlation map and protein-protein interaction networks were used to probe into the functional inclination of proteins and aberrated molecular networks in PDR vitreous. In addition, peptide-centric analysis of the proteome data was carried out to identify proteolytic processing, primarily ectodomain shedding events in PDR vitreous. Functional validation experiments were performed using preclinical models of ocular angiogenesis.

**Results:** The vitreous proteome landscape revealed distinct dysregulations in several metabolic, signaling, and immune networks in PDR. Systematic analysis of altered proteins uncovered specific impairment in ectodomain shedding of several transmembrane proteins playing critical roles in neurodegeneration and angiogenesis, pointing to defects in their regulating sheddases, particularly ADAM10, which emerged as the predominant sheddase. We confirmed that ADAM10 protease activity was reduced in animal models of ocular angiogenesis and established that activation of ADAM10 can suppress endothelial cell activation and angiogenesis. Furthermore, we identified the impaired ADAM10-AXL axis as a driver of retinal angiogenesis.

**Conclusion:** We demonstrate restoration of aberrant ectodomain shedding as an effective strategy for treating PDR and propose ADAM10 as an attractive therapeutic target. In all, our study uncovered impaired ectodomain shedding as a prominent feature of PDR, opening new possibilities for advancement in the DR therapeutic space.

## Introduction

Diabetic retinopathy (DR) is the most common microvascular complication of diabetes and the leading cause of vision impairment in working adults 1, 2. Duration of diabetes and severity of hyperglycaemia are major determinants underlying the development of DR 3, 4. The estimated global prevalence of DR among patients with diabetes is 35% affecting more than 100 million people worldwide 5, 6. DR manifests as vascular abnormalities in the retina characterized by capillary occlusion and weakening, increased vascular permeability, microaneurysm formation, and intraretinal hemorrhages in the early phase termed non-proliferative DR (NPDR) 1, 2, 7. These vascular changes lead to tissue hypoxia in the affected region which triggers the expression of a large number of hypoxia-responding factors, such as vascular endothelial growth factor (VEGF) 8, 9. As a consequence, new blood vessels form in an attempt to ameliorate the hypoxic condition and retore tissue perfusion. At the advanced stage of DR, known as proliferative DR (PDR), new and abnormal blood vessels start to form on the surface of the retina, and bleed into the vitreous cavity leading to vision impairment 10, 11. As the disease progresses, fibrotic scar tissue may form causing tractional retinal detachment and eventually blindness 11. Diabetic macular edema (DME) is a related form of vision-threatening stage of DR marked by swelling of the macula arising from the breakdown of the blood-retinal barrier which ultimately results in vision loss 12, 13.

Suppressing neovascularization and blood vessel leakage with VEGF inhibitors are the standard of care for PDR and DME 14-16. Despite the great efficacy, 30-50% of patients with PDR or DME do not respond well or may develop resistance to anti-VEGF therapy 2, 15. Such an inadequate response to anti-VEGF drugs suggests the involvement of additional molecular players other than VEGF in aggravating the complications of DR 2, 12. Indeed, inhibitors for other regulators of angiogenesis such as PDGF, Tie2, and PIGF have been tested in clinical trials, especially in individuals who are refractory to current anti-VEGF therapy 17-20. Effective targeted therapy addressing these clinical gaps in DR therapeutics is currently not available. This is mainly because of gaps in systemic understanding of molecular aberrations in DR, warranting interrogation of such molecular networks 2.

Besides angiogenic growth factors, other proteins such as hormones and enzymes are released by the injured retina in PDR and likely contribute to fibrovascular progression of the disease. Damages to the ocular tissues accompanying onset and progression of DR will impact the recruitment, shedding or turnover of receptors, the activation of downstream signaling transducers, and the release of target proteins into the vitreous humor. Such cellular and molecular changes can specifically occur during neovascularization or neuronal injury in the retina upon DR onset and development. We postulated that such alterations in the damaged ocular tissues will be reflected in the composition of the vitreous and hence underlying mechanisms and aberrant molecular pathways in PDR can be reconstructed through systematic analysis of dysregulated proteins in PDR vitreous. Quantitative mass spectrometry (MS)-based proteomics is one of the robust technologies to profile such dysregulated proteins in clinical samples as it enables identifying and quantifying thousands of proteins in an unbiased manner 21-23. Hence, here we employed this advanced technology to quantify the proteome of vitreous humor from patients with PDR in comparison to individuals with epiretinal membrane (ERM) without DR or other active retinal vascular disease as controls. ERM is avascular and does not accompany any vascular changes or modulation of vasculature-specific markers, and hence represents a suitable category of control for extracting novel angiogenic drivers and potential therapeutic targets in PDR, which is highly vascular. To this end, we carried out extensive interrogation of altered protein networks to unveil aberrated pathways associated with dysregulated vitreous proteome in PDR. Through this analysis, we identified several molecular players and their aberrations contributing to neuronal, vascular, and inflammatory responses accompanying PDR. Most importantly, we observed specific impairment in ectodomain processing and shedding in PDR patients as a prominent feature by analysing their vitreous profiles and identified reduced levels of several shed proteins that play key roles in various neuronal and vascular contexts in the PDR vitreous in comparison with the control. We revealed potential proteases responsible for such shedding events as plausible targets, of which we validated ADAM10 as an important player in modulating retinal angiogenesis using ocular-specific experimental models.

## Results

### Comprehensive vitreous proteome profiling

**Figure 1:**
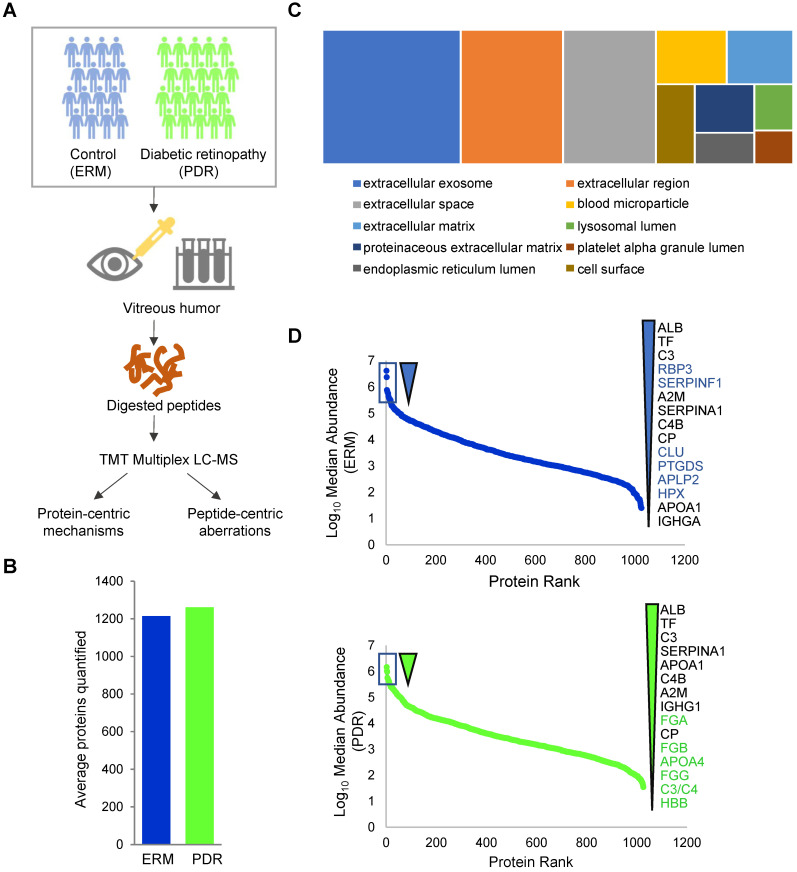
**Deep proteome profiling of PDR vitreous. (A)** Vitreous proteome profiling workflow. **(B)** Comparison of average quantified proteins in the PDR and control ERM sample groups. **(C)** Cellular component distribution of quantified vitreous proteins. **(D)** Ranked protein abundances in PDR and control ERM groups based on median vitreous protein abundance distribution within the groups. The top 15 most abundant proteins are highlighted.

To obtain a comprehensive mechanistic perspective underlying PDR, we performed a case-control study comprising of 20 PDR patients that represented the diseased condition and 20 individuals without PDR, with ERM, a proliferation of retinal glial and pigmented epithelial cells, as the control group. All the ERM cases used as control in this study were primary ERM cases, and not related to other secondary causes such as retinal vascular disease, retinal breaks, or previous laser treatments. The demographics of all participants used for the discovery cohort are described in Table 1 (Supplementary Table S1A). The vitreous humor samples from these 40 individuals were collected using approved clinical procedures with informed consent. Proteins were extracted from these samples (two samples were excluded at this stage, see methods) followed by trypsin digestion as described in the methods. For robust quantification of proteins across the samples, we adopted a tandem mass tag (TMT)-based approach for multiplexed quantification of proteins coupled with iso-electric focusing-based offline peptide fractionation to enhance overall peptide identification, and thus proteome coverage. The study pipeline is depicted in Figure 1A. At a 1% false discovery rate (FDR), we identified a total of 1805 proteins in total across all the samples (Supplementary Table S2 and Supplementary Figure S1A). The high coverage can be attributed to both the robust proteomic workflow as well as the wide sample cohort used in this study. This constitutes a comprehensive proteome coverage of the human vitreous, with a considerable portion of proteins consistent with those previously reported 24-27. On an average, we quantified about 1240 proteins per vitreous sample with approximately similar numbers (~1200 proteins) across both the ERM and PDR study groups, after excluding two outlier samples (Figure 1B and Supplementary Figure S1B). Notably, we obtained completeness in quantification for 847 proteins across all samples, and 1028 proteins were quantified in at least 75% of each individual group. Since vitreous proteins are extracellular and possibly secreted from surrounding eye tissues, we also assessed the quantified proteins for their cellular localization (Figure 1C). We observed that a majority of these proteins were predominantly extracellular, with terms such as 'extracellular exosome', 'extracellular space' and 'extracellular region' showing high enrichment. Apart from these, we also observed enrichment for 'blood microparticle'. These could possibly be explained by the highly angiogenic nature of the retina during retinopathy. In addition, we also observed a proportion of proteins encompassing other cellular compartments including cell surface and lumen indicating the involvement of secretory proteins, and platelet alpha-granules rich in growth factors and coagulation factors.

We next investigated the abundance spectrum of proteins across the ERM and PDR groups, which revealed that the quantified proteins spanned over a wide range of magnitude across both the groups (Figure 1D). A closer look into the top abundant proteins highlighted several proteins including albumin, transferrin, complement protein C3 and α1-antitrypsin (SERPINA1) as consistently abundant among both the control and PDR groups. Certain abundant factors in ERM vitreous such as RBP3, CLU, SERPINF1, PTGDS, APLP2 and HPX, however, occupied lesser abundance niches in the PDR group. Among these, the retinol binding protein 3 (RBP3) is considered a prognostic marker offering protection against the development of DR 28, 29, clusterin (CLU) is an anti-permeability factor for the treatment of diabetic blood-retinal barrier breakdown causing vascular leakage 30, and SERPINF1 (PEDF) is regarded as a suitable candidate for gene therapy for neovascularization due to it mediating anti-angiogenic and anti-fibrotic effects 31, 32. All these suggest a loss of protective factors in the vitreous of PDR patients that complicates DR severity. Such a loss could possibly arise from aberrations in protein secretions or selective degradation of the abundant proteins. In contrast to the control group, PDR vitreous showed higher abundance of proteins that constitute the fibrinogen complex (FGA, FGB, FGG) known to promote retinal fibrosis, ultimately leading to PDR development 33. Thus, the overall vitreous abundance landscape captured is consistent with physiological changes during PDR.

**Figure 2:**
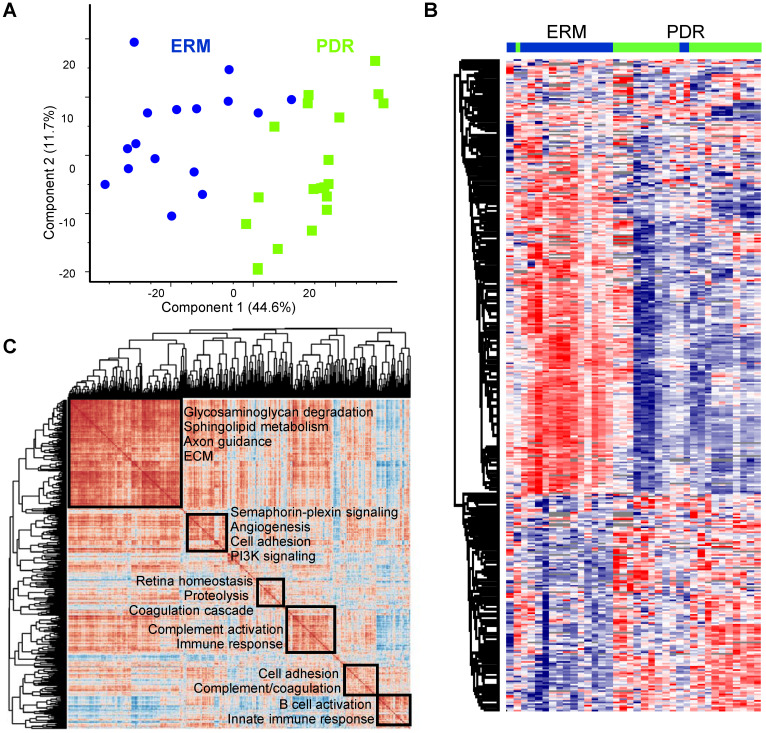
**Proteome landscape of PDR vitreous. (A)** Principal component analysis of PDR and ERM samples based on protein expression. **(B)** Hierarchical clustering of samples based on z-score normalized relative abundance is shown as a heatmap. **(C)** Global correlation map of all proteins across the PDR samples by assessing the Pearson correlation coefficients of all protein combinations. Highly correlated protein clusters are highlighted along with their functional annotation terms. Positive correlations are indicated by deep brown and negative correlations are shown as blue.

With top abundant proteins showing stark differences among the two groups, we next assessed whether the PDR vitreous profiles were globally distinct from the control ERM group. After Z-scoring relative abundances across the groups, we observed that the PDR and the ERM groups showed clear stratification according to the disease status along the first component, suggesting that the vitreous landscape is unique to the group with its constituents altered with disease status (Figure 2A). Of the four ERM samples that were also diabetic (one excluded due to technical reasons, see methods), three clearly separated out from the PDR samples and clustered along with the ERM samples that were non-diabetic, and only one sample was in the borderline between the PDR and ERM grouping (Supplementary Figure S1C). Hierarchical clustering among all the samples distinctly revealed two clusters and highlighted reduced expression of ~2/3^rd^ of the proteins specifically in the PDR group compared to the control ERM group (Figure 2B). Such an extensive remodeling of the vitreous with PDR is indicative of loss of possible protection factors and anti-angiogenic components, and reflects on distinct molecular aberrations of neighboring ocular tissues undergoing disease-specific rewiring.

### Proteome landscape of PDR vitreous

To gain molecular perspective on the functional inclination of PDR vitreous we interrogated global co-regulations among all quantified proteins within the group. From the correlation map encompassing about a million protein comparisons, the PDR vitreous highlighted six predominant clusters (Figure 2C). The largest cluster included proteins mainly involved in axon guidance and extracellular matrix (ECM) organization. Apart from these neuronal and structural components, metabolic proteins involved in sphingolipid metabolism and glycosaminoglycan degradation were also enriched within this cluster. Another correlation cluster included signaling proteins functioning in the semaphorin-plexin and PI3K pathway that is regarded as a promising target for ocular neovascularization 34. Apart from these, we identified several clusters of proteins that function in various aspects of coagulation cascade and complement activation. The control group also revealed functionally distinct correlated clusters with the major cluster showing preferential enrichment for nervous system development and axon guidance (Supplementary Figure S2A). In contrast to the PDR group, the semaphorin signaling components maintained high correlation with this primary cluster in the control group thus aligning with their functional inclination in mediating neuronal processes. In addition, the control ERM group also revealed clusters pertaining to metabolic pathways such as gluconeogenesis and IGF signaling as against the PDR group. This is indicative of key perturbations in abundances and possible associations of metabolic proteins with DR development. From the control correlation map, we also observed tighter co-regulation among several proteins involved in the coagulation and complement cascade to form a major cluster in comparison to several smaller clusters observed in PDR. This finding iterates that selective complexes or groups of proteins within these cascades are differently modulated in terms of their abundances and associations leading to DR. More importantly, the identification of neuronal proteins in both the PDR and control vitreous highlights that key neuronal proteins originating in eye components such as retina, ciliary body or choroid can accumulate in vitreous, and thus can shed insights on molecular aberrations accompanying pathological processes.

### Functional alterations in PDR vitreous

**Figure 3:**
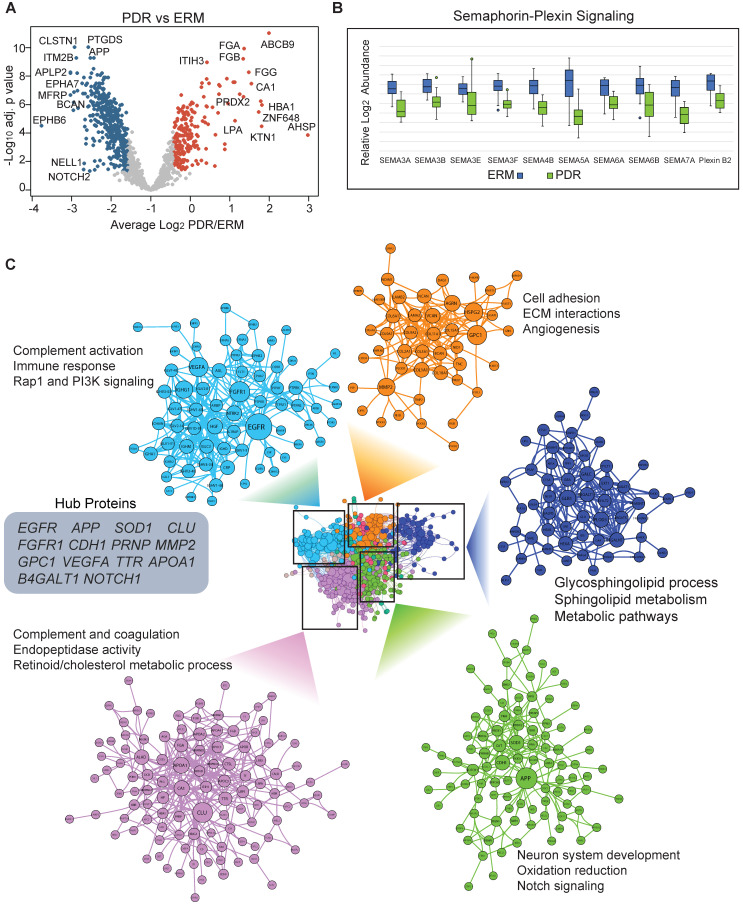
**Altered proteome profiles in PDR vitreous. (A)** Volcano plot showing differentially expressed proteins (adj. p < 0.05 and 1.5-fold change). Red and blue represent proteins showing increased and decreased abundance in PDR vitreous. Top differentially expressed proteins are highlighted. The PDR overexpressed proteins are indicated by red and underexpressed proteins are indicated by blue. **(B)** Boxplot showing protein abundance levels of semaphorin signaling components. **(C)** Vitreous protein interaction network of PDR-altered proteome is shown. Densely connected protein modules extracted from the network are highlighted along with their functional annotation. Node sizes correspond to the number of associations shared by the protein with its neighbors. Hub proteins based on network centrality is shown within grey box.

We next assessed vitreous proteome alterations associated with PDR and found that a total of 620 proteins were altered significantly (adj. p < 0.05). As observed in the global proteome profile, a majority of these altered proteins were downregulated (413) and only 207 proteins showed specific upregulation in the PDR group at about 1.5-fold change (Figure 3A and Supplementary Table S3A). The upregulated proteins were primarily involved in complement activation, innate immune response and coagulation cascades which concurs with known phenotypic features in PDR individuals. Enzymes such as carbonic anhydrase 1 (CA1) known to mediate vascular permeability and retinal hemorrhage, and multiple proteins involved in lipoprotein metabolism and cholesterol efflux also showed overexpression in PDR vitreous 35. Serum lipoprotein(a) which is regarded as a predictor of diabetic microvascular complications was observed to be highly expressed in the vitreous of PDR patients 36. Among the several apolipoproteins in serum that have been previously correlated with DR progression and prognosis 37, we observed some of these including APOA1/A2/A4/C1/C3 to be significantly overexpressed in PDR vitreous. The exact origin of these proteins, how these proteins end up in the vitreous, and whether they are from surrounding tissues such as the retina, or infiltrating serum proteins is however unclear. Nevertheless, these proteins have the potential to be genuine biomarkers given their modulation in both vitreous and serum during DR progression. The downregulated proteins on the other hand spanned a multitude of processes including neuronal, metabolic, signaling and morphogenetic. Several of these such as NOTCH2, APP, ITM2B and PTGDS have been implicated in neurological disorders. We also observed enzymes functioning in sphingolipid metabolism and semaphorin signaling to be selectively under-expressed in PDR vitreous (Figure 3B). In addition to inhibiting VEGF functions, semaphorin components, particularly semaphorin 3A (SEMA3A) has been reported to reduce pathological vascular modulations in preclinical model of retinal neovascularization 38. These proteins we found in the vitreous may have likely originated from the retina as semaphorins play crucial roles in establishing synaptic connections, neuronal migration, and circuit assembly. Thus, our observations of modulated proteins predominantly associated with neuronal or vascular defects are consistent with release of cellular proteins into the vitreous by surrounding damaged tissues accompanying disease state either by vascular leakage, loss of structural integrity, or regulated shedding. We also performed a similar analysis excluding the few 'diabetic' ERM samples from the ERM group, and this revealed significant alterations of 608 proteins (at adj. p < 0.05 and 1.5-fold change) with 587 common proteins between the two analyses in comparison with PDR (Supplementary Figure S2B and Table S3B). Interestingly, no significant protein alterations were found between the ERM groups based on their diabetic status (Supplementary Table S3C). This affirms that the few ERM samples with diabetes retain similar proteome profiles as their non-diabetic counterpart and hence do not pose any bias in the comparative analysis.

Next, we explored functional connectivity among the altered proteins in PDR to unravel their involvement in molecular rewiring (Figure 3C). For this, we assembled a protein-protein interaction network that represented both physical and functional associations such as those altering phosphorylation, regulating expression or degradation and complex assembly (Supplementary Table S4). Though the interaction network may not entirely represent molecular remodeling accompanying retinopathy in dysregulated tissue/cells except for those that occur extracellularly involving proteases acting on specific extracellular/membrane targets or receptor-target engagement, it could recapitulate those interactions occurring in surrounding tissues such as retina, choroid or ciliary body due to release or leakage into the vitreous. The assembled network revealed a dense topology indicative of close-kinit associations among the PDR-modulated proteins. We identified distinct modules showing specific functional inclinations. The top module with high number of connected proteins included many of the overexpressed proteins and showed preferential enrichment of complement and coagulation cascade. One of the top abundant proteins in control vitreous, and also the most connected protein clusterin (CLU), however, is downregulated in PDR implying defects in its associated networks. The next PDR-modulated cluster mainly involved proteins functioning in neuron system development, oxidation reduction and notch signaling among other pathways. This cluster included several proteins such as APP and APLP1, and the notch receptors NOTCH1/2/3 involved in amyloid fiber formation and its associated processes. From the network, we established that proteins involved in angiogenesis functionally interacted with ECM, and this is consistent with reports implicating ECM remodeling and degradation with angiogenic activity 39. The network also revealed several functional hubs including APP, SOD1, NOTCH1, VEGFA, FGFR1 and CLU that are crucial in effectively relaying signal during PDR-induced network modeling. These hub proteins encompassed various functions including growth factor signaling, synapse formation, neural development and proteolysis emphasizing intricate molecular rewiring during PDR.

A closer look at the proteins in the PDR functional network revealed that regulated proteolytic processing underlies several of these accompanying processes. For instance, presinilin pathway involving the gamma-secretase complex cleaves many surface-anchored proteins to induce various signal transduction events including notch signaling 40. Amyloid precursor protein (APP) that emerged as a key hub in the PDR vitreous network is proteolytically cleaved differentially to induce either the amyloidogenic or non-amyloidogenic pathway dictating neurodegenerative/protective effects 41. Notably, we also found sheddases, which cleave transmembrane protein substrates to release their extracellular proteolytic fragment, such as ADAM9 and ADAM22, within the same APP containing cluster. Extracellular proteins such as collagens are proteolytically activated to form collagen fibers that extensively remodel matrix behavior, and regulated functional cleavage of chondroitin sulfate proteoglycans such as vesrican (VCAN) and brevican (BCAN) is implicated in neural plasticity 42. Notably, the complement system components also co-clusters with these proteolytic processing proteins along with other signaling pathways such as PI3K signaling and cholesterol metabolism. This is indicative of crosstalk between complement components and some of these pathways which are linked to various neuronal and vascular responses. Thus, from the PDR altered network it is evident that apart from physical interactions among the proteins, regulated functional interactions underlying proteolytic processing may also accompany DR development to shed different substrate proteins into the vitreous.

### Impaired ectodomain shedding in PDR

**Figure 4:**
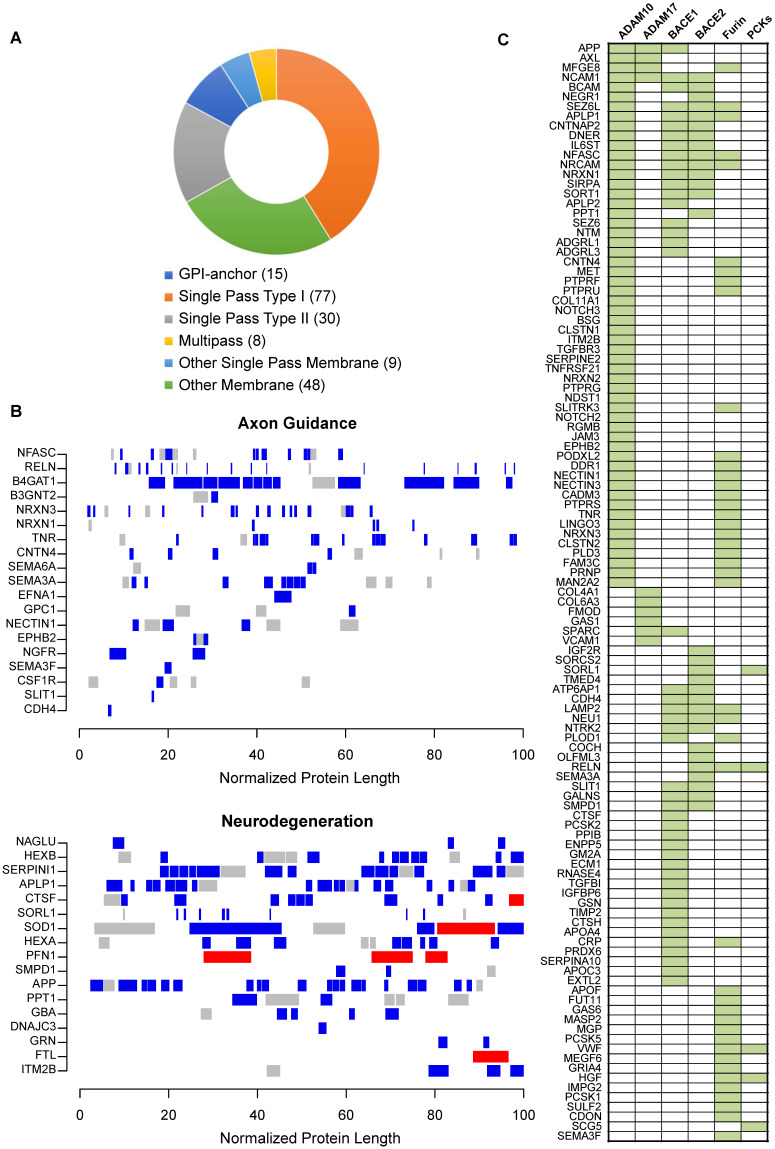
** Impaired ectodomain shedding in PDR. (A)** Topological analysis of predicted cleaved and shed substrates in the vitreous. **(B)** Proteins displaying differential proteolytic cleavage across the PDR and the control groups are shown. Peptides shown in blue and red indicate reduced or increased abundance by 1.5-fold in the PDR group, and those in grey indicate no change in peptide levels between the two groups. The protein lengths are normalized to a scale of 100 for visualization. **(C)** Sheddase mapping of the predicted substrates is visualized as a heatmap. Green indicates that the protein is a known substrate of the corresponding sheddase.

Our functional network analysis indicated that dysregulated protein networks in PDR are associated with sheddase activities. Sheddases, which are transmembrane proteases, cleave extracellular (ecto) domains of transmembrane proteins, releasing soluble ectodomains into extracellular space. Tightly regulated sheddase activities are crucial for generation of proteolytic fragments or neo-proteins that elicit paracrine signaling or activation of downstream signaling from cleaved intracellular protein domains such as those with NOTCH or other receptor tyrosine kinases such as AXL 43, 44. Manual analysis of our proteomics results indicated downregulation of some ectodomains in PDR vitreous in comparison to control ERM group. For example, mapping of identified peptides of APP showed that level of α-APP, an ectodomain released from APP by sheddase α-secretase activity, was low in PDR vitreous. These observations led us to hypothesize that relative differences in peptide amounts arising from potential proteolytic cleavage will be preserved in the peptide quantification information and extracting such inherent differences would shed insights on altered proteolytic events in a global manner. Accordingly, we strategized a peptide-centric mapping approach to assess distributional differences along the length of the protein to extract all possible protein cleavage events (Supplementary Table S5). To account for cleavages that occur at both termini along different lengths within the protein, we also analysed multiple blocks of varied terminal lengths and assessed for significant differences in peptide distributions (p < 0.05 and 1.5-fold change) within each varied terminal block among the groups. Thus, using this approach we were able to distinctly isolate those proteins with inherent distributions in peptide patterns in the vitreous as against those proteins that are only differentially secreted, and accordingly predicted 444 substrates to undergo differential proteolytic cleavage (Supplementary Table S6). Analysis of the predicted substrate proteins revealed that many were extracellular secreted (189) and membrane bound (186). Topological classification of the substrates revealed a strong enrichment for single pass type-I membrane proteins (such as APP) which serve as preferential substrates for ectodomain shedding events (Figure 4A). Single pass type-II membrane and GPI-anchored proteins accounted for a total of 30 and 15 proteins, respectively, while only a small proportion represented multipass membrane proteins.

The predicted substrates included the well-documented APP along with several other membrane receptors such as NOTCH, EPHB2 and ITM2B that are known to be proteolytically cleaved (Supplementary Figure S3). We also identified bona fide APP-like proteins, APLP1 and APLP2, that are cleaved by various secretases to release their ectodomains. Functional mapping of these substrates emphasized their involvement in various neurologically relevant processes including axon guidance, neurodegeneration and also angiogenesis that contributes to vascular damage in DR (Figure 4B and Supplementary Figure S4). Among the substrates processed under axon guidance are neurexins (NRXN1 and NRXN3) and SLIT1 involved in synapse formation, semaphorins (SEMA3A and SEMA6A), nectin (NECTIN1) associated with synapse adhesions and nerve growth factor (NGFR) stimulating nerve growth and differentiation of neurons.

Next, we investigated if these predicted substrates are associated with specific upstream proteases or sheddases. Since many of the predicted substrates undergo ectodomain shedding we focused on substrates of α- or β-secretase, along with proprotein convertase family of proteins such as furin that are capable of processing ectodomains from membrane proteins. Apart from substrates curated in protease-related databases like MEROPS, we additionally curated the literature for large-scale substrate screening experiments primarily involving secretome-based proteomic profiling in the context of protease knockdown/knockout model 45. With the multitude of information, we observed that as many as 57 substrates are bona fide ADAM10 substrates, which is an α-secretase (Figure 4C). These include APP, AXL, APLP1/2, ITM2B, and several receptor tyrosine phosphatase family members such as PTPRF, PTPRU, PTPRS and PTPRG. Several of these have been reported to maintain retinal functions including APP which is expressed in neuronal cells of the inner retina and involved in inner retinal circuitry 46, and APLP2 whose deletion induced retinal synaptopathy and retinal degeneration 47. In addition, presynaptic NRXN1, NRXN2 and NRXN3, postsynaptic membrane proteins calsyntenins (CLSTN1 and CLSTN2), L1 family of neuronal cell adhesion molecule NRCAM, neurite outgrowth regulating SLITRK3, all were identified to be substrates of ADAM10. This reiterates that key neuronal processes including synapse formation and assembly and neuron-neuron adhesion are impacted during retinopathy and accompanying vascular changes, and these are evident with altered vitreous composition in PDR. In addition to ADAM10, we also observed several substrates of BACE1 with altered proteolytic patterns. These include known targets such as SEZ6, SEZ6L, and latrophilins (ADGRL1/3). Whether the activity of both ADAM10 and BACE1 are impaired in PDR, or if the molecular aberrations in one of these proteases propagates and impacts the other, causing neuronal defects in the retina or other affected ocular tissues, is however unclear.

### Reduced ectodomain shedding of ADAM10 substrates in PDR

**Figure 5:**
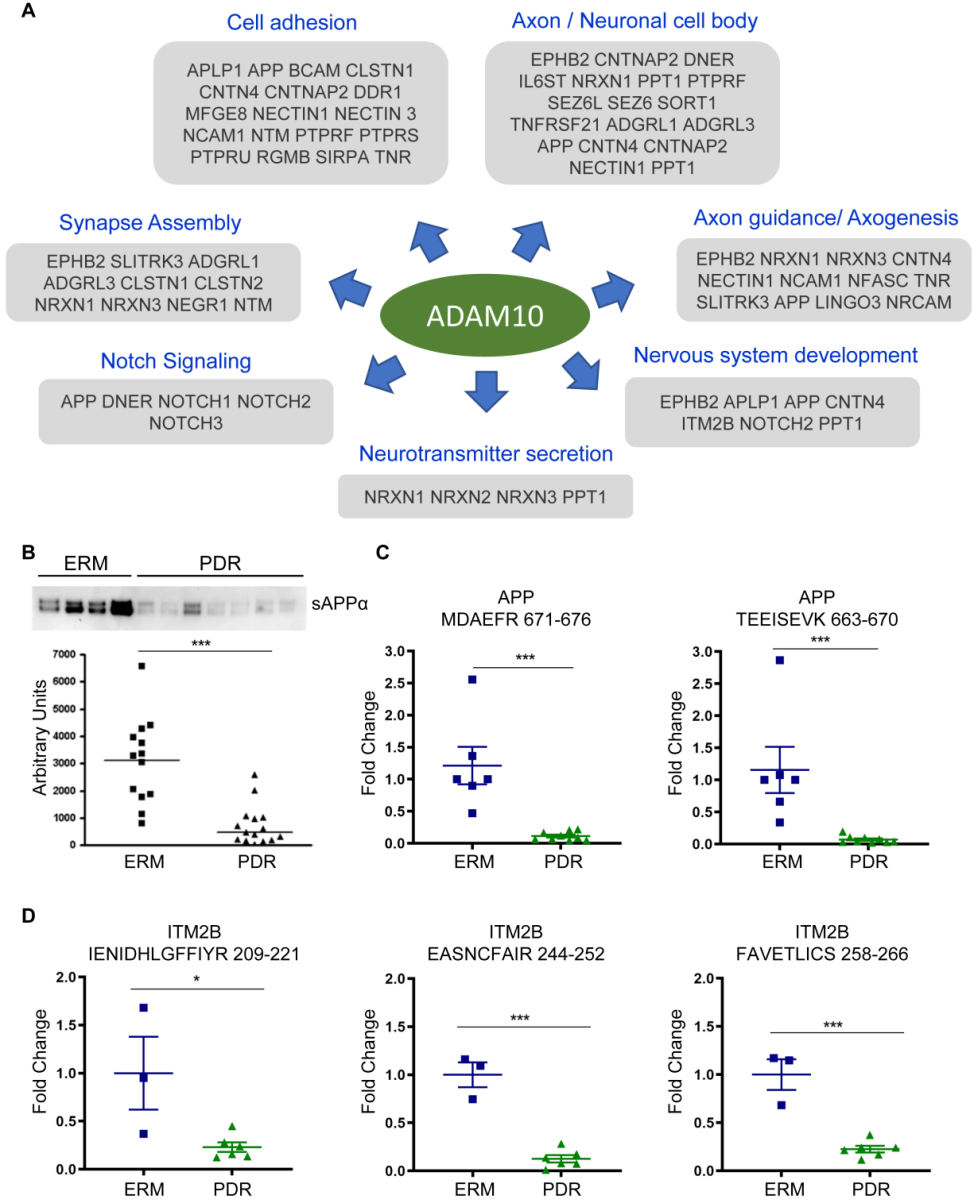
** Shedding of ADAM10 substrates impaired in PDR. (A)** Overview of selected ADAM10 substrates found to be modulated in PDR vitreous and their respective functional involvement is shown. **(B)** Western blot analysis of sAPPα in ERM and PDR sample groups. Representative blot is shown. Data for the plot are presented as mean ± s.e.m. Statistical analysis was determined by unpaired, two-tailed Mann Whitney test; *** p < 0.001 **(C)** Targeted proteomics assay to monitor specific peptides in the fragment cleaved by ADAM10 within the ectodomain region of APP. Data are presented as mean ± s.e.m. Statistical analysis was determined by unpaired, two-tailed Mann Whitney test; * *p* < 0.05, ** p < 0.01, and *** p < 0.001. **(D)** Targeted proteomics assay to monitor specific peptides generated by sequential cleavage of ITM2B in the ectodomain region. Peptide 209-221 is obtained by ADAM10 cleavage following prior cleavage by furin to generate fragments containing the other two indicated peptides. Data are presented as mean ± s.e.m. Statistical analysis was determined by unpaired, two-tailed Mann Whitney test; * *p* < 0.05, ** p < 0.01, and *** p < 0.001.

With ADAM10 identified as the predominant sheddase implicated in the proteolytic processing of several predicted substrates, we focused on ADAM10-mediated events to unravel the pathology associated with PDR disease state. Many of the ADAM10 substrates are important constituents of axon or neuronal cell body, and impact diverse neurological phenomenon including axogenesis, neurotransmitter secretion and synapse assembly (Figure 5A). For some substrates, we also identified site-specific cleaved peptides generated by ADAM10 in the vitreous proteome (Supplementary Figure S5A). For instance, we identified the peptide corresponding to α-cleavage site (ADAM10) at position 688 and found it to be downregulated by over 3-fold. It is interesting to note that while APP can also be processed by β-secretase BACE1, we did not identify the peptide corresponding to this cleavage. Instead, we identified a peptide spanning this cleavage site but showing no BACE1-specific cleavage (BACE1 cleavage should happen in the position after the methionine in the indicated peptide at position 672). This iterates that the proteolytically shed APP that we identified in the vitreous arises from ADAM10-specific cleavage rather than BACE1. ITM2B also known as Bri2, which inhibits amyloidogenic processing of APP to form toxic amyloid-beta protein 42 (Aβ42) oligomers, also showed reduced levels of specific peptide cleaved by ADAM10. ITM2B undergoes sequential processing by furin followed by ADAM10 to release the brichos domain 48. Our MS data revealed that both the peptide cleaved by furin as well as spanning the brichos domain (peptide 209-221) were reduced in abundance in PDR in comparison to control ERM individuals suggesting a defect in ITM2B processing by ADAM10. For the APP like proteins APLP1 and APLP2, we identified peptides resulting from both ADAM10 and BACE1 cleavage 49. For APLP1, ADAM10 and BACE1 can target the same site and hence there is a possibility that these substrates are cleaved by both the sheddases to varying extents in different tissues 50. To identify the possible localization of these substrates, we mapped these proteins to those identified previously in several eye tissues including retina, RPE choroid, ciliary body and iris (Supplementary Figure S5B). While some of the proteins were found to be expressed in all tissues, a few were exclusively found only in the retina or RPE choroid. We found that a few substrates including APLP2 and ITM2B can be derived from retina. The phosphatases, PTPRU, PTPRS and PTPRG, are predominantly localized in the RPE choroid.

The reduced shedding of ADAM10 substrates specifically in PDR implied a defect in ADAM10 protease activity and its processing during retinal angiogenesis and DR onset. In order to verify our hypothesis, we performed Western blot analysis of sAPPα in an-independent cohort of vitreous samples (Supplementary Table S1B) and confirmed the reduction of sAPPα generated by ADAM10 significantly in the PDR group compared to the control ERM group (Figure 5B). We performed targeted proteomics assay by parallel reaction monitoring (PRM) to specifically monitor the unique peptides MDAEFR and TEEISEVK of APP derived by cleavage by ADAM10 and not BACE1. This also confirmed significantly reduced levels of the cleaved α-peptide in APP in the PDR group compared to the other group (Figure 5C). We extended this assay to monitor another predicted ADAM10 substrate ITM2B and observed reduced levels of representative peptide (at position 209-221 amino acid) from its brichos domain fragment generated by ADAM10 (Figure 5D and Supplementary Figure S6). In addition, we also noticed reduced levels of peptides that are pre-processed by furin prior to cleavage by ADAM10 (position 244 onward). Taken together, these data provide evidence that processing of ADAM10 substrates is impaired in PDR, implying reduced enzymatic activity of ADAM10 during neovascularization and development of DR.

### Reduced ADAM10 activity in mouse models of ocular angiogenic diseases

**Figure 6:**
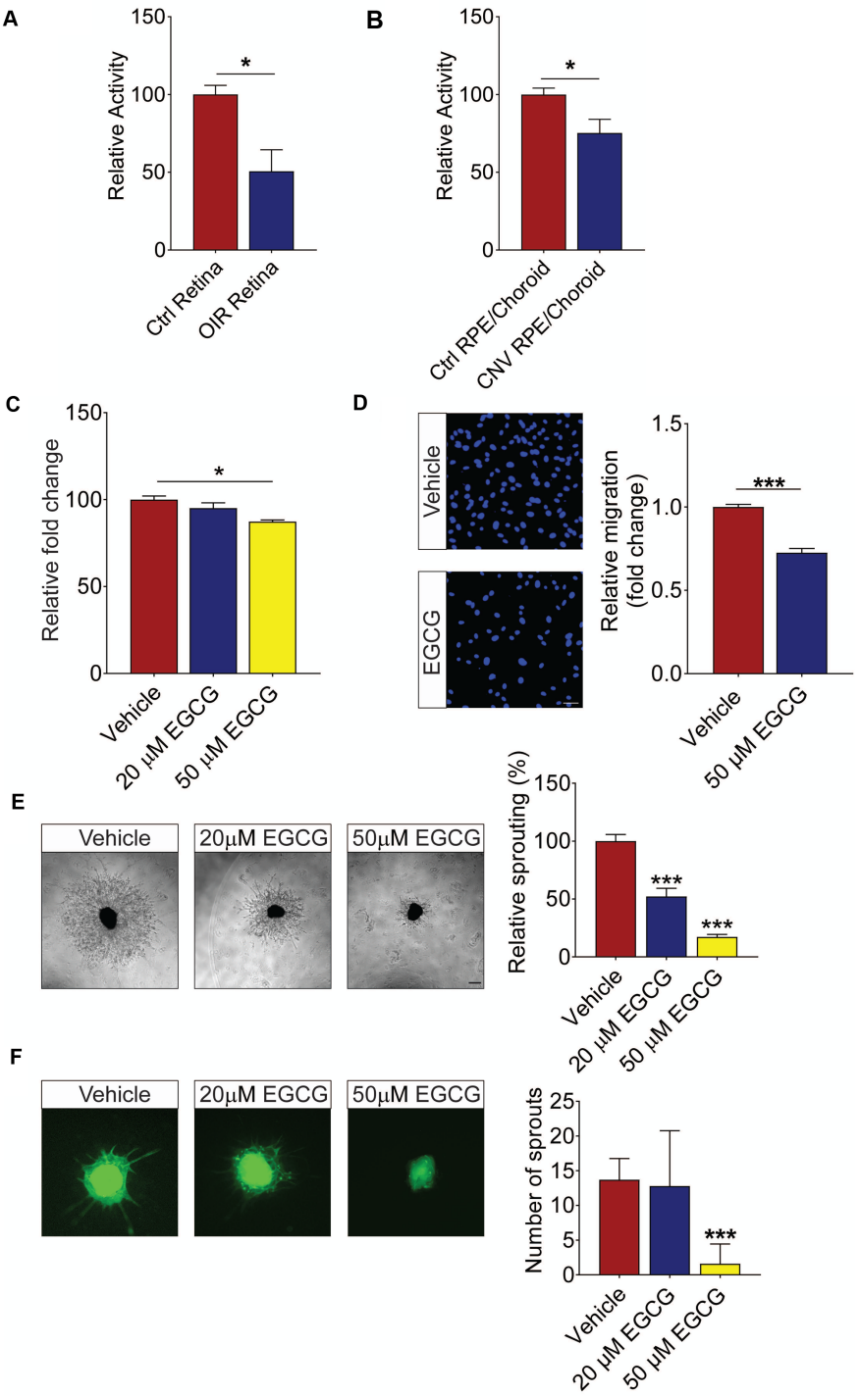
** ADAM10's activator EGCG inhibits the activation of human retinal endothelial cells (HRECs) *in vitro* and vessel outgrowth in *ex vivo* models. (A and B)** ADAM10 activity impairment in ocular disease models are shown.** (A)** ADAM10 activity in retinae from control mice and OIR mice. n = 3. **(B)** ADAM10 activity in choroid/RPE from control mice and CNV mice. n = 4. Data are presented as mean ± s.e.m. Statistical analysis was determined by unpaired, two-tailed Student's *t*-test; * *p* < 0.05. **(C)** AlamarBlue assay demonstrated inhibition of HRECs viability by 50 µM EGCG following 24 h treatment (n = 3). **(D)** DAPI staining demonstrated inhibition of HRECs migration by 50 µM EGCG following 4 h treatment (n = 3). **(E)** EGCG inhibits choroidal vessel outgrowth in a dose-dependent manner. Representative images (left) and quantitative analysis (right) of microvessel formation from mouse choroidal explants demonstrating a significant inhibitory effect of EGCG (n = 3 independent experimental groups, n ≥ 6 explants per treatment group). **(F)** EGCG inhibits aortic vessel sprouts at the dosage of 50 µM. Representative images (left) and quantitative analysis (right) of macrovessel formation from mouse aortic explants demonstrating a significant inhibitory effect of EGCG (n = 3 independent experimental groups, n ≥ 6 explants per treatment group). Scale bar: 100 µm. All images shown are representative, and data are presented as mean ± s.e.m. Statistical significance was determined by one-way ANOVA followed by Tukey's multiple comparison test or unpaired, two-tailed Student's *t*-test; * *p* < 0.05 and *** *p* < 0.001.

The analysis of vitreous proteins pointed to a defect in ADAM10 proteolytic activity in PDR patients. To confirm this hypothesis, we checked the activity of ADAM10 in two mouse models of ocular angiogenic diseases. First, we checked the ADAM10 activity in retinae collected from mice subjected to oxygen-induced retinopathy (OIR). This revealed that the activity of ADAM10 is reduced by around 50% in OIR retinae as compared to that in normoxic retinae, which is consistent with our observation made in PDR patient samples (Figure 6A). We further observed around 30% reduction in ADAM10 activity in the choroid/RPE compartment of the mouse eye subjected to laser-induced choroidal neovascularization (CNV) (Figure 6B). Such an impaired activity of ADAM10 could result from maturation defects such as pro-domain cleavage necessary for its enzymatic activation. The proteolytic activity of ADAM10 is regulated by its pro-domain which functions as a specific inhibitor of its activity and subsequent shedding events 51, 52. Indeed, our MS data also revealed downregulation of ADAM10 pro-domain trimming proteases including PCSK2 and PCSK5 in PDR vitreous. From this evidence, we infer that reduced ADAM10 activity in PDR is likely due to diminished pro-domain trimming.

### ADAM10 activator inhibits angiogenesis

Having demonstrated the reduced activity of ADAM10 in ocular tissues of mouse models of ocular angiogenesis, we went to test whether the activation of ADAM10 could suppress endothelial cell activation and angiogenesis using various human retinal endothelial cells (HREC)-based *in vitro* models and *ex vivo* models of angiogenesis. Epigallocatechin-3-gallate (EGCG), a green tea polyphenol, was previously demonstrated to elevate active ADAM10 levels and ADAM10-dependent shedding has been shown to attenuate TNFα-induced endothelial cell activation 53, 54. Here we showed that 50 µM EGCG led to around 15% reduction in HREC proliferation following 24 h treatment (Figure 6C). Similarly, 50 µM EGCG significantly suppressed the migration of HRECs across the Transwell by ~30% (Figure 6D). Angiogenesis is a complex process involving extensive interaction between different types of cells and extracellular matrix components 55. To probe angiogenesis in a multicellular environment, we next investigated whether EGCG could inhibit blood vessel outgrowth in various *ex vivo* models of angiogenesis. We first subjected mouse aortic rings to treatment with 20 µM and 50 µM EGCG, respectively. Our result demonstrated that 50 µM EGCG significantly inhibited VEGF-induced aortic ring sprouting (Figure 6E). We next assessed EGCG's effect on ocular-specific angiogenesis in mouse choroidal sprouting assay. We observed that EGCG demonstrated a dose-dependent inhibition on choroidal vessel outgrowth as compared to the vehicle-treated controls (Figure 6F). Taken together, these data showed a strong inhibitory role of EGCG on retinal endothelial cell activation and neovascularization from different vascular beds in a dose-dependent manner.

### Impaired ADAM10-AXL axis triggers retinal angiogenesis

**Figure 7:**
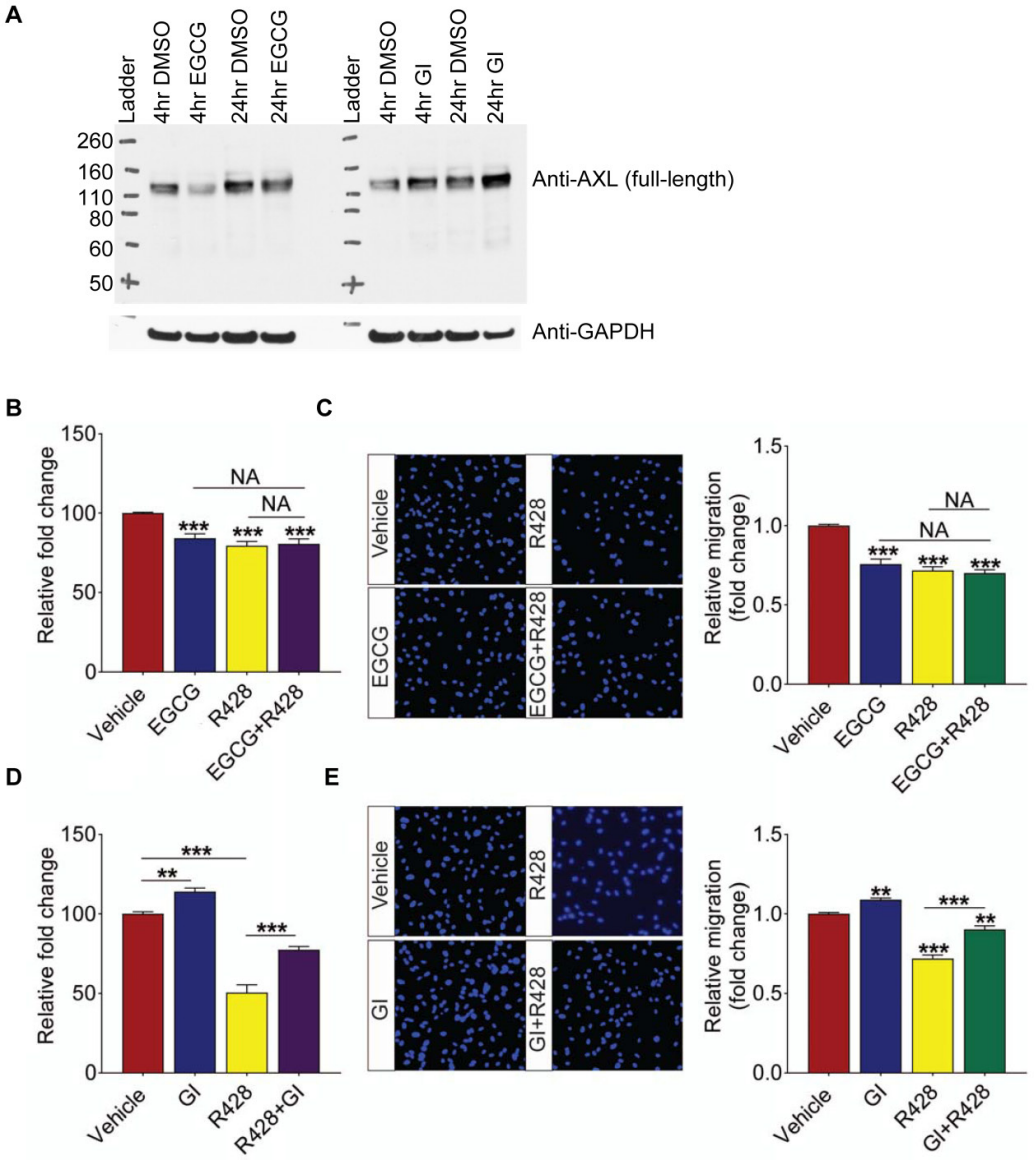
** AXL is ADAM10's downstream substrate and ADAM10's function is dependent on the activity of AXL. (A)** Full-length AXL protein levels in HREC with 50 µM ADAM10 activator EGGC or 10 µM ADAM10 inhibitor GI 254023X is shown at both 4- and 24-h post treatment. GAPDH is used as loading control** (B)** Combination treatment of AXL inhibitor R428 and ADAM10 activator EGCG have no additive effects on HRECs viability. AlamarBlue assay demonstrated inhibition of HRECs viability by 50 µM EGCG, 1 µM R428 or combination treatment of 50 µM EGCG, 1 µM R428 following 24 h treatment (n = 3). **(C)** Combination treatment of AXL inhibitor R428 and ADAM10 activator EGCG have no additive effects on HRECs migration. DAPI staining demonstrated inhibition of HRECs migration by 50 µM EGCG, 5 µM R428 or combination treatment of 50 µM EGCG, 5 µM R428 following 4 h treatment (n = 3). **(D)** R428 reverses the promoting effect of EGCG inhibitor GI 254023X on HRECs viability. AlamarBlue assay demonstrated effects of HRECs viability by 10 µM GI 254023X, 1 µM R428 or combination treatment of 10 µM GI 254023X, 1 µM R428 following 24 h treatment (n = 3). **(E)** R428 reverses the promoting effect of GI 254023X on HRECs migration. DAPI staining demonstrated effects of HRECs migration by 10 µM GI 254023X, 5 µM R428 or combination treatment of 10 µM GI 254023X, 5 µM R428 following 4 h treatment (n = 3). Scale bar: 100 µm. All images shown are representative, and data are presented as means ± s.e.m. Statistical significance was determined by one-way ANOVA followed by Tukey's multiple comparison test; ** *p* < 0.01 and *** *p* < 0.001.

Having established the impaired activity of ADAM10 in ocular angiogenic models and showing potent anti-angiogenic effect of ADAM10 activator EGCG *in vitro* and *ex vivo*, we next investigated whether ADAM10 exerts its effect by regulating ectodomain processing of its substrates. A known ADAM10 substrate, AXL, is an important mediator of tumour angiogenesis 56, 57. Impaired shedding of AXL was also observed in DR vitreous in our proteomic profiling. To assess if ADAM10 activity impacted the level of full-length AXL protein due to cleavage and release of ectodomain in retinal endothelial cells, we treated HRECs with ADAM10 activator EGCG for 4 h and 24 h and assessed for protein levels of full-length AXL by Western blotting. This revealed that EGCG-induced ADAM10 activation reduced full-length AXL protein levels and this effect was more pronounced in the 4 h treatment than in the 24 h (Figure 7A). Conversely, treatment with ADAM10 inhibitor GI254023X, elevated the protein levels of full-length AXL at both 4- and 24-h post treatment. Taken together, this indicates that ADAM10 can accelerate proteolytic cleavage of AXL, resulting in reduced levels of full-length AXL that can impact AXL-induced downstream signaling events. Having established that ADAM10 regulates AXL levels, we next investigated whether dysregulated ADAM10-AXL axis is involved in triggering retinal angiogenesis. For this, we treated HRECs with ADAM10 activator EGCG, AXL inhibitor, R428, or the combination of both compounds. We observed that inhibitory effect of EGCG on HREC proliferation was comparable in all treatment groups (Figure 7B). Consistent with this observation, no additive effect was observed for EGCG and R428 in HREC migration (Figure 7C), suggesting that ADAM10 inhibits angiogenesis through shedding of its downstream substrate, AXL. To further validate our hypothesis, we next checked whether R428 could antagonize the pro-angiogenic effects of an ADAM10 inhibitor, GI254023X. This revealed that GI254023X promoted HRECs' proliferation by about 15% and the pro-proliferating effect of GI254023X was significantly attenuated by R428 (Figure 7D). Similarly, the promoting effect of GI254023X on HREC migration was also strongly attenuated by R428 (Figure 7E). In all, our data showed that the activation of ADAM10-AXL axis exhibited potent anti-angiogenic effects.

## Discussion

Current treatment for ocular angiogenic diseases is dominated by VEGF-based therapeutics and anti-VEGF drugs that have led to unprecedented improvements in vision preservation and quality of life 1, 2, 10, 15. Despite their great efficacy, anti-VEGF drugs are not universally effective, likely due to the compensatory activation of alternative angiogenic pathways following the suppression of VEGF signaling. Unraveling non-VEGF drivers responsible for ocular angiogenesis is important for developing alternative or complementary treatments to anti-VEGF drugs. The onset and progression of DR arises from a cascade of biochemical and molecular events resulting in extensive structural remodeling of retina and the tissues around it 58. Vitroretinal relationships have been long established for several ocular pathologies including DR, and vitreous components such as cytokines correlating with PDR severity and degree have been previously derived 59. This suggests that local changes in the retina and vitreous both determine the level of dysfunction arising from DR, and the clinical manifestations of DR such as either detachment or hemorrhage vastly influence the composition of vitreous. In fact, VEGF was first discovered from PDR vitreous previously, which led to the development of anti-VEGF therapy that has completely transformed the clinical management of DR 60. In this regard, we explored the vitreous proteome profiles of PDR patients and controls (patients with diabetes without PDR with ERM, and non-diabetic individuals with ERM) to derive significant alterations accompanying DR. Apart from the aberrated molecular networks and signaling pathways, one of the key findings from our study was the identification of impaired ectodomain shedding in PDR, which has not been revealed in any previous studies thus far. Our analysis uncovered that ectodomain shedding events are specifically impaired in PDR patients based on reduced abundances of several proteolytic substrates playing fundamental roles in neural circuitry. Importantly, we demonstrated that ADAM10 is a crucial sheddase showing reduced activity in PDR and that restoring ADAM10 activity by activator EGCG can strongly inhibit retinal angiogenesis. While there have been studies previously focusing on vitreous proteomes to understand PDR, these do not provide comprehensive in-depth investigations for novel advancements in the PDR therapeutic space. Proteolytic processing and ectodomain shedding are key cellular processes accompanying several neuronal and vascular modulations, and providing a holistic snapshot of potentially altered ectodomains and linking them to their impaired upstream protease for novel targeting has not been discussed before in light of retinal angiogenic diseases.

Our in-depth vitreous proteome landscape revealed various functional clusters that are modulated during PDR. Overall, we found that complement and coagulation systems are specially overexpressed in PDR vitreous. The complement system functions as a bifunctional switch in modulating inflammatory response in several ocular diseases including DR and age-related macular degeneration, and abnormal activation of complement components accompany the progression of these diseases 61. While the mechanisms underlying neuropathy and vasculopathy in DR are yet unclear, significant involvement of inflammatory mediators has been reported by several groups. Chronic retinal inflammation is one of the prime pathogenic factors in DR causing both neuronal and vascular damage, and several studies have shown infiltration by circulating immune cells promoting such a degenerative response 62. In fact, some of these complement factors such as C5a have been known to induce the release of IL-6 and VEGF exacerbating the disease pathology 63, 64. With an upregulation of key complement components including C3 and CFH by at least 1.4-fold, our data concur with an overall activation of the alternative complement pathway in PDR 65. Importantly, both C4A and C4B that are central to classical and lectin pathways were observed with no significant changes in PDR, and complement factor I (CFI), a major negative regulator of complement pathways, with alternative pathway being the most affected, showed reduced abundance in PDR. This, in addition to increased protein abundance of membrane attack complex (MAC) components (C5, C8 and C9 at >1.5-fold and C6 and C7 at ~1.3-fold), the accumulation of which has been reported in the choriocapillaris of patients with DR 66, corroborates the involvement of complement pathway activation during DR evoking a plethora of pathological sequelae.

In addition to the immune modulators, several signaling components also showed modulation in the PDR vitreous. Secreted semaphorins are key functional molecules regulating axonal and dendrite outgrowth and hence serve as guidance cues for nervous system development. The observance of several of the semaphorin family of proteins downregulated in PDR is indicative of significant impairment or deterioration of neural establishments, which could ultimately result in retinal neurodegeneration. In fact, in RPE cells, SEMA3A reduced migratory and proliferative effects of RPE in addition to inhibiting VEGF utilization, and hence proposed as a promising treatment candidate for RPE proliferative diseases 67. In the retina, SEMA3A has been shown to inhibit pathological vascular changes during neovascularization 38. In contrast, some studies have reported SEMA3A to be induced in early phases of DR and cause vascular permeability 68. While a disease stage-specific modulation of semaphorin molecules is possible, our data is consistent with reduction in overall semaphorin signaling.

Beyond altered protein levels, by looking deeper into the peptide profiles of vitreous from PDR and control groups, we uncovered possible defects in ectodomain shedding during DR. Ectodomain shedding is a key process regulating intercellular communication and shedding of cleaved proteolytic fragments impact a wide array of cellular process including protein targeting, signaling, angiogenesis, and cell death 44. While the impact of ectodomain shedding is widely appreciated in the context of neurological disorders such as Alzheimer's their relative importance in ocular pathologies remains underappreciated 69. In line with APP fragments that are regarded as potential biomarkers, the cleaved protein targets we have identified in the context of PDR have prospects to serve as functional biomarkers for disease diagnosis or even prognosis. The impaired ectodomain shedding in PDR implicated key sheddases such as ADAM10, and even BACE1 to be modulated in DR, and several of the ADAM10 substrates have also been reported to be cleaved by β-secretase, particularly BACE1 including the three APP family of proteins 70, 71. This points to a plausible crosstalk between the two sheddases for cleavage of particular substrates, which may also be dependent on the cellular context. We uncovered several substrates of ADAM10 including APP and its associated proteins, NOTCH receptors, and other proteins regulating neurite outgrowth and assembly, such as IgLON family protein neuronal growth regulator 1 (NEGR1), and neurotrimin (NTM) with dysregulated processing in PDR vitreous implicating aberrations in ADAM10 activity in DR. On the immune front, IL-6, a potent proinflammatory cytokine has been reported to be elevated in PDR and is one of the prime mediators of retinal vascular inflammation during PDR 72. The key signaling transducer that aids IL6 signaling, interleukin-6 receptor subunit beta (IL6ST or GP130), has been observed with reduced shedding in PDR vitreous. Shedding of this transducer has been attributed to ADAM10 and shed forms of IL6ST have been shown to impede hyper IL6-induced trans-signaling 73, 74. Besides this, ADAM10 also cleaves a wide range of substrates to regulate their turnover. For instance, ADAM10 mediates the shedding of TNFα-receptor I (TNF R1) and the soluble TNF RI acts as a TNF antagonist that can inhibit TNFa-mediated proinflammatory effects 75, 76. All these evidences potentially link impaired ADAM10 activity with increased inflammation and vascular injury during DR.

The metalloprotease ADAM10 has major developmental roles and is associated with both normal and pathophysiological events. ADAM10 is an essential gene and is important for shedding several proteins involved in brain development, and in fact, ADAM10 null nice die early at the embryonic stage due to defects in the development of the central nervous system, somites, and vascular complications 77. In fact, ADAM10 represents the most dominant α-secretase in the brain playing central roles in various neuronal developmental processes. In the developing retina, deficiency of ADAM10 induces cone and ganglion cell differentiation along with depletion of progenitor cells and abnormal neurogenesis, hence considered indispensable for retinal development 78. In addition to its neuronal roles, ADAM10 has also been reported to impact angiogenic sprouting 79. All these evidences suggest that ADAM10 potentially regulates a spectrum of functions in ocular tissues and perturbations to its activity manifest as retinal degeneration and neovascularization.

Through our systematic proteomic MS strategies, we propose restoration of aberrant ectodomain shedding as an attractive therapy for PDR. Indeed, our subsequent experimental validation showed that ADAM10 activation by EGCG strongly inhibits endothelial cell activation and angiogenesis. Besides EGCG, there are various commercially available ADAM10 enhancers/activators, of which Acitretin (ADAM10 enhancer) is an FDA-approved drug for psoriasis and is also now under phase II clinical trial for Alzheimer's disease. These activators can be tested for their efficacy in inhibiting pathological neovascularization in PDR once the role of ADAM10 in pathological angiogenesis is functionally validated using *in vivo* preclinical models of ocular angiogenesis. Furthermore, we showed that AXL inhibition is required for the action of ADAM10. Therefore, it is possible to target AXL directly also as a treatment for ocular angiogenic diseases. Besides PDR, abnormal blood vessel formation is a characteristic feature for ocular angiogenic diseases, such as neovascular age-related macular degeneration (nAMD) and retinopathy of prematurity (ROP). Therefore, it will be interesting to explore the efficacy of ADAM10 activation or AXL inhibition in other ocular diseases as well.

One of the major limitations of our study is the use of ERM samples as a control group to extract PDR-specific processes and pathways. While both PDR and ERM represent pathological conditions with gliosis, ERM is avascular in nature with very few or no blood vessels. This aligns well with our interest in investigating PDR which is a highly vascular disease marked by the expression of vascular-specific markers and neovascularization causing leaky blood vessels. In the lack of possibility to obtain vitreous samples from a healthy control population, ERM as a suitable alternative control has been used in several previous studies such as ours 24, 25, 80. Nevertheless, we have validated the key finding, impaired ADAM10 activity in the context of retinal angiogenesis, through relevant preclinical models of ocular angiogenesis and *in vitro* functional assays, that provide orthogonal confirmation to inferences we draw from proteomic profiling. Another limitation is that the mouse models of OIR and laser-induced CNV are acute models of angiogenesis and may not fully recapitulate the progressive vascular damage and long-standing immune dysregulations in human patients with DR. However, both these preclinical models exhibit excessive ocular angiogenesis and have been extensively used for evaluating novel therapeutic target discovery and drug screening for such ocular angiogenic diseases as PDR 81-84, and hence is suitable for our reported work. In the current work, we have profiled the vitreous from PDR to recapitulate potential upstream mechanisms in contrast to assessing the actual ocular tissues. When procuring actual tissues may be a challenge, profiling liquid biopsy samples in close association to the tissues of defect is more likely to offer new knowledge on several such diseases. Similar studies to extrapolate on disease mechanisms and uncover possible novel markers have also been carried out on cerebrospinal fluid to gain insights into neurodegenerative diseases such as Alzheimer's or Parkinson's 85, 86. Lastly, while in this study we have profiled a cohort of samples representing the PDR group, it would be worthy to explore a larger cohort of patients with varying stages of the disease to holistically represent the heterogeneity of PDR, considering PDR is a highly progressive disease.

Taken together, our study proposes that restoring aberrant ectodomain shedding in PDR by targeting respective sheddases with impaired activities would prove as a prudent approach for designing effective therapies for PDR. This opens a new arena for targeted therapies for retinal angiogenesis which becomes urgent in the lack of efficacy with current treatments, particularly for anti-VEGF non-responders.

## Materials and Methods

### Human vitreous sample collection

The study was performed according to the guidelines set out in the WMA Declaration of Helsinki and approved by the local institutional review board, Singhealth cIRB 2015/2672. The study cohort included a total of 40 patients including 20 with PDR and 20 ERM individuals as controls. Patients with PDR without gross vitreous hemorrhage who required vitreoretinal surgery due to tractional retinal detachment were recruited for the discovery cohort. Controls were recruited from patients requiring vitreoretinal surgery due to significant visual impairment from ERM and had no observable active retinal vascular disease. Of these 5 had diabetes and 15 were not diabetic. The baseline characteristics of the PDR and ERM patients are summarized in Table 1 and included in Supplementary Table S1A. An independent validation cohort was used for subsequent validation experiments and the corresponding clinical demographics data is included in Supplementary Table S1B. Written informed consent was obtained before trans pars plana vitrectomy during which vitreous samples (500 μl) were collected at the start of the surgery via aspiration using a vitreous cutter with the infusion off to avoid dilution of the sample. Samples were immediately placed on ice and transferred to the laboratory under cold chain. The samples were centrifuged at 4°C to remove any cellular debris and then stored at -80 °C degrees until proteomics analysis.

### Sample preparation for mass spectrometry

Stored vitreous samples were thawed on ice and centrifuged to remove any remaining debris. The samples were then resuspended with 9 M urea/100 mM ammonium bicarbonate (Sigma-Aldrich Pte. Ltd.) buffer. Protein concentration was determined using Bradford Assay (Bio-Rad Laboratories) after dilution to 3 M urea. Two samples (ERM33 and ERM38) were excluded at this stage due to low protein concentration. For all the other samples (n = 18), 100 µg from each sample were then subjected to reduction with dithiothreitol (Sigma-Aldrich Pte. Ltd.) (final concentration of 5 mM) for 30 min at room temperature, alkylation with iodacetamide (Sigma-Aldrich Pte. Ltd.) (final concentration of 10 mM) for 30 min at room temperature in the dark, followed by overnight digestion with 1 µg of LysC (Lysyl Endopeptidase, Wako) at 37 °C. Subsequently, 50 mM ammonium bicarbonate was added to each sample to adjust the urea concentration to 1 M before adding 2 µg of Sequencing Grade Modified Trypsin (Promega). The samples were incubated for 8 h at 37 °C followed by desalting with the C18 solid phase extraction cartridge (3M company) and drying under vacuum to ~40 µL.

### TMT 10-plex labeling

Desalted peptides were subjected to tandem mass tag (TMT) labeling using TMT10plex™ Isobaric Label Reagent Set (Thermo Scientific™) according to the manufacturer's protocol. All samples were run in a total of five sets and samples were assigned to each TMT set by randomization, in addition to pooled control and pooled PDR samples. Briefly, TMT label was reconstituted in 41 µL anhydrous acetonitrile (Sigma-Aldrich) before mixing with respective peptides and incubated for 1 h. Reaction was quenched using final 5% hydroxylamine (Merck) in triethylammonium hydrogen carbonate buffer (Fluka) for 15 min. Labeled peptides were mixed before vacuum-dried and fractionated using off-gel isoelectric focusing.

### Off-gel isoelectric focusing

Agilent 3100 OFFGEL Fractionator (Agilent, G3100AA) was used to fractionate the labeled peptides into 24 fractions according to manufacturer's protocol. The fractions were desalted using self-packed C18 (3M company) stage tips and vacuum-dried prior to mass spec analysis.

### Liquid chromatography and mass spectrometry

For proteome analysis, peptides were reconstituted in 0.1% formic acid for analysis on Thermo Easy nLC 1000 that was connected to Orbitrap Fusion™ Tribrid™ Mass Spectrometer (Thermo Scientific™). The trap column used was C18 Acclaim PepMap™ 100 of 3 μm, 100 A, 75 μm I.D. x 2 cm nanoViper and the analytical column was PepMap™ RSLC C18, 2 μm, 100 A, 75 μm I.D. x 50 cm. The LC solvent A comprised of 0.1% formic acid in 2% acetonitrile and LC solvent B comprised of 0.1% formic acid in 95% acetonitrile. The gradient was as follows: 8 - 40% solvent B in 180 min; 40 - 100% solvent B in 10 min; 100% solvent B for 10 min at the flow rate of 200 nL/min. The mass spectrometer was set in the data dependent acquisition mode. Full scan MS spectra (m/z 310 - 1510) were acquired with a resolution of R = 120,000 at an AGC target of 4e5 and a maximum injection time of 50 ms. MS2 orbitrap HCD scan of resolution of 60,000 with AGC target of 1e5 and fragmented using a normalized collision energy of 34% with fixed first mass at 120 and isolation window of 1.2 m/z.

### MS data processing

For 10plex-TMT labeled proteome analysis, raw MS data was processed using Proteome Discoverer 2.1 (Thermo Scientific™). Database search was performed using the integrated Sequest HT search engine against the Uniprot human FASTA database (release 2017) for tryptic peptides with maximum of two missed cleavage sites, MS and MS/MS mass tolerance of 10 ppm and 0.02 Da, respectively. Searches included cysteine carbamidomethylation and TMT-modifications at peptide N-termini and lysine residues as fixed modifications, and protein N-terminal acetylation and methionine oxidation as dynamic modifications. Peptide and protein identifications were performed at false discovery rate (FDR) < 0.01.

### Bioinformatics data analyses

Protein quantifications obtained from individual PDR and ERM samples were used for all downstream analyses. The 126 TMT-reporter channel consisting of pooled samples included in each TMT set was used as a reference for normalization across all five TMT proteome datasets and the normalised protein abundance for each protein was expressed as protein ratio over the abundance of the reference channel. The Perseus software package versions 1.6.14 and 1.6.15 were used for proteome data post-processing 87. Protein ratios were log2 transformed for all subsequent data analysis. Only those proteins quantified in at least 75% of the samples within each group were considered. Two samples, ERM29 and ERM35, were removed from further analysis as they were identified as outliers based on principal component analysis. Principal component analyses and hierarchical clustering analyses were performed on z-score transformed protein ratios. For generation of protein abundance profile plots, median normalized protein abundances across all samples within each group were used. The median abundances within each group were visualized as log10-transfomed abundances. For correlation profile map analysis, all pairwise protein-protein correlations were calculated using Pearson correlation method and a correlation cluster map was generated using the resulting correlation coefficients. The correlation calculation, map generation (packages: gplots and RColorBrewer) and cluster extraction (package: dendextend) were all performed in R statistical environment. Gene ontology and pathway enrichment analysis of the identified proteins within relevant clusters were assessed using DAVID and enriched ontology and pathway terms were extracted (p < 0.05). For differential expression analysis of proteins, limma analysis was carried out as implemented in the Bioconductor package “limma”. Those proteins that were significantly altered at adjusted p value < 0.05 with at least 1.5-fold change were regarded as differentially expressed. The differentially expressed proteins were visualized as volcano plot using R statistical environment.

### Protein network analysis

Functional connectivity between significantly altered proteins were explored using protein-protein interactions as curated in the Pathway Commons and Omnipath database that represent comprehensive data resources compiling pathway, interaction and signaling data from multiple sources 88, 89. For construction of interaction network, in addition to those direct physical interactions among proteins, functional associations encompassing those that regulate expression, controls protein state change such as phosphorylation, catalysis or mediate complex formation were also considered. The overall functional network spanned interactions representing metabolic, signaling a well as regulatory pathways. All duplicate interactions were removed to reduce redundancy. The network was visualized using Gephi and module function using default parameters was applied to extract clusters. The functional biases of the identified clusters for ontology and pathways were derived from DAVID. Finally, topological parameters of the network were assessed for node centrality and degree distribution using Cytoscape.

### Protease substrate tracking

For cleaved protein tracking, in-depth peptide analyses to map conserved peptide distributional differences along the total lengths of proteins were performed. To account for possible neo-peptides arising from protein cleavages, MS data was re-processed in the semi-tryptic mode, and all peptides including semi-tryptic peptides identified and quantified from the proteome profiling of PDR and control ERM samples were used. For further analysis, only peptides quantified in over 50% of samples within each group were retained. Differential modulation of individual peptides within a protein in the PDR group as against the ERM control group was assessed using Student's t-test. To accommodate for possibility of cleavage at either termini (N or C terminal) and at different lengths of the protein, the entire analysis was iterated through different lengths of cleaved protein fragments. As such the analysis considered a starting 5% terminal region at the N-terminal and incremented the terminal length by 1% at each iteration ending at 95% to cover potential N- and C-terminal cleavages along the length of the protein. At each iteration, the proportion and conserved differences in peptide amounts in a subset of such terminal peptides as against the rest of the peptides quantified in the protein was measured as a factor. Proteins with multiple peptides modulated significantly at 1.5-fold at p < 0.05 in the specific iterated terminal length as against the total peptides quantified were considered putative target substrate proteins with altered proteolytic cleavage patterns. Membrane protein topology information were obtained from Uniprot and the identified substrate hits were matched against this database to identify the topological nature of the predicted substrate hits. Curated upstream proteases for the predicted substrates were obtained from MEROPS peptidase database 45, and additional information on protease-substrate relationships were obtained from literature, specifically from large-scale MS proteomic screens aimed at identifying modulated substrates upon protease knockdown/inhibition. Data were gathered from several MS studies pertaining to modulation of ADAM10, ADAM17, BACE1, BACE2 and Furin. The predicted substrate pool was matched against the database of literature-curated protease-substrate relationships to map potential upstream proteases for the predicted ectodomain shedding protein.

### Parallel Reaction Monitoring

PRM analysis was carried out for selected peptides from ectodomain region of APP and ITM2B in vitreous samples. Briefly the digested peptides from each sample were analysed on Orbitrap Fusion™ Tribrid™ Mass Spectrometer (Thermo Scientific™) interfaced with EASY-nLC 1000 nanoflow liquid chromatography system (Proxeon, Fisher Scientific). The digested peptides were reconstituted in 0.1% formic acid and separated on an analytical column (PepMap™ RSLC C18, 2 μm, 100 A, 75 μm I.D. x 50 cm). The LC solvent A comprised of 0.1% formic acid in 2% acetonitrile and LC solvent B comprised of 0.1% formic acid in 95% acetonitrile. Selected PRM amenable peptides were monitored using a targeted inclusion list. One microgram of peptides was separated on an analytical column at a flow rate of 200 nL/min at 45°C using a 95 min gradient: 0 to 34% LC solvent B for the first 70 min, followed by a 10 min gradient ranging from 34 to 100% LC solvent B and maintained at 100% LC solvent B for 15 min. Targeted-MS2 scans were acquired with a resolution of 30,000, an AGC target of 1e5 with a maximum injection time of 105 ms. The mass range of m/z 100 - 2000, an isolation window of 1.2 m/z with 25% collision energy were used. The raw data were subsequently analysed using Skyline v3.1.0.7382 (http://skyline.maccosslab.org).

### Animals

Male and female C57BL/6J mice were purchased from Invivos (Singapore) and were kept on a 12 h light-dark cycle and fed a standard rodent chow (NCD, 18% kcal from fat, Harlan). *Ex vivo* assays, oxygen-induced retinopathy mouse model and laser-induced choroidal neovascularization mouse model, which require animals, were performed in compliance with the guidelines of the Institutional Animal Care and Use Committee of Singapore Eye Research Institute (SERI) (2020/SHS/1597) and the Association for Research in Vision and Ophthalmology Statement for the Use of Animals in Ophthalmic and Vision Research.

### Materials, cells, and cell culture

SensoLyte® 520 ADAM10 Activity Assay Kits were purchased from Anaspec (Fremont, CA, USA). (-)-Epigallocatechin gallate (EGCG) and GI254023X were purchased from Sigma (St. Louis, MO, USA). R428 was purchased from Selleckchem (Houston, TX, USA). Primary human retinal endothelial cells (HRECs) were purchased from Angioproteomie (Boston, MA, USA) and maintained in endothelial growth media 2 (EGM2), which contained EBM2 basal media and Endothelial Cell Growth Medium BulletKits^TM^ (Lonza, Basel, Switzerland) or endothelial basal media 2 (EBM2), which contained 0.2 % FBS according to the supplier's instruction.

### Mouse model of oxygen-induced retinopathy (OIR) and laser-induced choroidal neovascularization (CNV)

OIR was induced as described 90. In brief, P7 mice and nursing mothers were placed in a 75% oxygen 12 chamber for 5 days before being returned to room air. After OIR mice have been returned to room air for 5 days (P17), the retinae of OIR mice and their age-matched mice were collected for ADAM10 activity analysis. CNV was induced as described 84. At 14-day post laser induction, mice were sacrificed and choroid/RPE plexus was separated from the neuroretina for ADAM10 activity analysis.

### AlamarBlue Cell Proliferation Assay

A total of 4x10^3^ HRECs were cultured in either EGM2 media or EBM2 media containing 0.2% FBS with different combination of treatments indicated in figure legends for 24 h. AlamarBlue (Life Technology, Carlsbad, CA, USA) solution was incubated with HRECs for 4 h to allow viable cells to be labeled. The colorimetric signal was captured by Infinite M200 Pro (Tecan, Männedorf, Switzerland). The relative viability of HREC in the treatment group was determined by normalizing the absolute absorbance reading from the compound-treated group to that of vehicle-treated control.

### Transwell migration assay

4×10^4^ cells subjected to respective treatments were seeded onto 8.0 μm pore size Transwell plates (Corning, NY, USA) and were allowed to migrate for 4 h at 37 °C. Migrated cells were fixed in 1% PFA and stained with 4′,6-diamidino-2-phenylindole (DAPI) (Sigma, St. Louis, MO, USA) before being visualized under the Zeiss AXIO (Zeiss, Oberkochen, Germany) and counted manually using ImageJ Cell Counter plugin.

### Aortic ring assay

Aortic ring assay was performed as described 91, 92. Aorta from the postnatal day 3 (P3) C57BL/6J mice was cut into 1 mm rings before being embedded in a 96-well plate coated with rat tail collagen I gel (BD Biosciences, San Jose, CA, USA). Explants were incubated for 30 min at 37 °C to allow for complete polymerization of the collagen gel. After incubation, aortic ring explants were cultured in 100 µL of OptiMEM supplemented with 2% FBS and 1× 1× penicillin and streptomycin overnight. The following day, media was replaced with OptiMEM supplemented with 2% FBS and 1× penicillin and streptomycin containing 50 ng/mL VEGF with different concentrations of EGCG, accordingly. Treatment media were changed every other day. At day 10 of culture, the explants were fixed in 4% PFA and stained with Griffonia Simplicifolia Lectin (GSL) isolectin IB4 (Vector Lab, ZB0406) and AN2-PE (Miltenyi Biotec, 130-100-468). Vessel outgrowth was visualized under the Eclipse Ti-E Inverted Research Microscope. The number of sprouts was counted manually.

### Choroid Sprouting Assay

Choroid sprouting assay was performed as described 92. Treatment media containing either vehicle or EGCG at concentration of 20 µM, and 50 µM were added to the explants on the day of explant embedding. Media were changed every other day. Images were taken after 4 days of treatment. Vessel outgrowth was visualized under the Eclipse Ti-E Inverted Research Microscope (Nikon, Tokyo, Japan). The sprouting area was quantified by TRI2 software (Version: 3.0.1.2, TRI2, Oxford, UK).

### Statistical Analysis

Data were presented as mean ± s.e.m. Statistical significance was performed using two-tailed, unpaired, Student's t-test when we compared the ADAM10 activity in retinae or choroid/RPE tissues from control and disease models and when we compared the migration rate of HRECs between vehicle group and EGCG-treated group. One-way ANOVA followed by a Tukey post-test analysis was used for the rest of the studies where the efficacy of multiple treatment groups was compared using GraphPAD Prism (Version: 7.04, GraphPAD Software Inc., San Diego, CA, USA). * p < 0.05; ** p < 0.01; *** p < 0.001. Each represents significant statistical comparisons among the listed (x-axis) experimental groups.

### Western blot analysis

Cells were lysed with 9 M urea lysis buffer (20 mM HEPES, pH 8.0, 9 M urea, 1 mM sodium orthovanadate, 2.5 mM sodium pyrophosphate, and 1 mM β-glycerophosphate) and protein amount were quantitated using Pierce™ 660nm Protein Assay Kit. Samples were then run on 4 - 12% Bis-Tris SDS-PAGE gel (Invitrogen) and transferred to a 0.2 µM PVDF membrane (Bio-Rad Laboratories). The membrane was blocked with 5% non-fat dried milk in TBST (20mM Tris, pH 8.0, 150 mM NaCl, 0.05% Tween 20) for 1 h before incubating with the respective primary antibody in TBST overnight at 4 °C on a roller. Subsequently, the membrane was subjected to washing with TBST for 5 min thrice; incubation with the respective secondary antibody in 5% non-fat dried milk in TBST for 1 h; washing with TBST for 5 min thrice; 1 min incubation with the ECL substrate (Immobilon Crescendo Western HRP substrate, MerckMillipore) and exposed to X-ray film for signal detection. AXL antibody was purchased from R&D systems (AF154) and GAPDH antibody from Santa Cruz Biotechnology (sc-32233). For vitreous analysis, soluble sAPPα fragment detecting antibody was purchased from IBL (11088).

## Supplementary Material

Supplementary figures and table legends.

Supplementary table 1.

Supplementary table 2.

Supplementary table 3.

Supplementary table 4.

Supplementary table 5.

Supplementary table 6.

## Figures and Tables

**Table 1:** Demographic description of patients

Cohort Description	ERM, *n = 20*	PDR, *n = 20*
Age	66 ± 5.5	55 ± 10.9
*Sex,* Male (n)	10	14
*Sex,* Female (n)	10	6
Right/Left eye (n/n)	10/10	12/8
IOP (mm Hg)^†^	15.7 ± 1.8	18.3 ± 4.3
HBA1c	*	8.1 ± 1.56

*HBA1c available only for 5 patients (mean = 6.42 ± 1.29); † Intraocular pressure in study eye.
