# Supplementary material for: System-wide vitreous proteome dissection reveals impaired sheddase activity in diabetic retinopathy
Source: Theranostics. 2022 Sep 11;12(15):6682–704. doi: 10.7150/thno.72947 (PMC9516227; doi:10.7150/thno.72947)
Supplement: Supplementary file 1 — Supplementary figures and table legends. [file thnov12p6682s1.pdf]

Supplementary Material for

System-wide vitreous proteome dissection reveals impaired sheddase activity in diabetic retinopathy

Asfa Alli-Shaik[1]†, Beiying Qiu[1,2,4]†, Siew Li Lai[1], Ning Cheung[2,3,4], Gavin Tan[2,3,4], Suat Peng Neo[1], Alison Tan[2,4], Chiu Ming Gemmy Cheung[2,3,4], Wanjin Hong[1], Tien Yin Wong[2,3,5], Xiaomeng Wang[1,2,4*], Jayantha Gunaratne[1,6*]

[1] Institute of Molecular and Cell Biology (IMCB), Agency for Science, Technology and Research (A*STAR), Singapore 138673.

[2] Singapore Eye Research Institute (SERI), Singapore 169856.

[3] Singapore National Eye Centre (SNEC), Singapore 168751.

[4] Centre for Vision Research, Duke-NUS Medical School, Singapore 169857.

[5] Tsinghua Medicine, Tsinghua University, Beijing China 100084.

[6] Yong Loo Lin School of Medicine, National University of Singapore, Singapore 117549.

† These authors contributed equally to this work.

*To whom correspondence should be addressed: Co-corresponding authors, jayanthag@imcb.a-star.edu.sg and xiaomeng.wang@duke-nus.edu.sg

This PDF file includes:

Figures S1 to S6
Tables S1 to S6

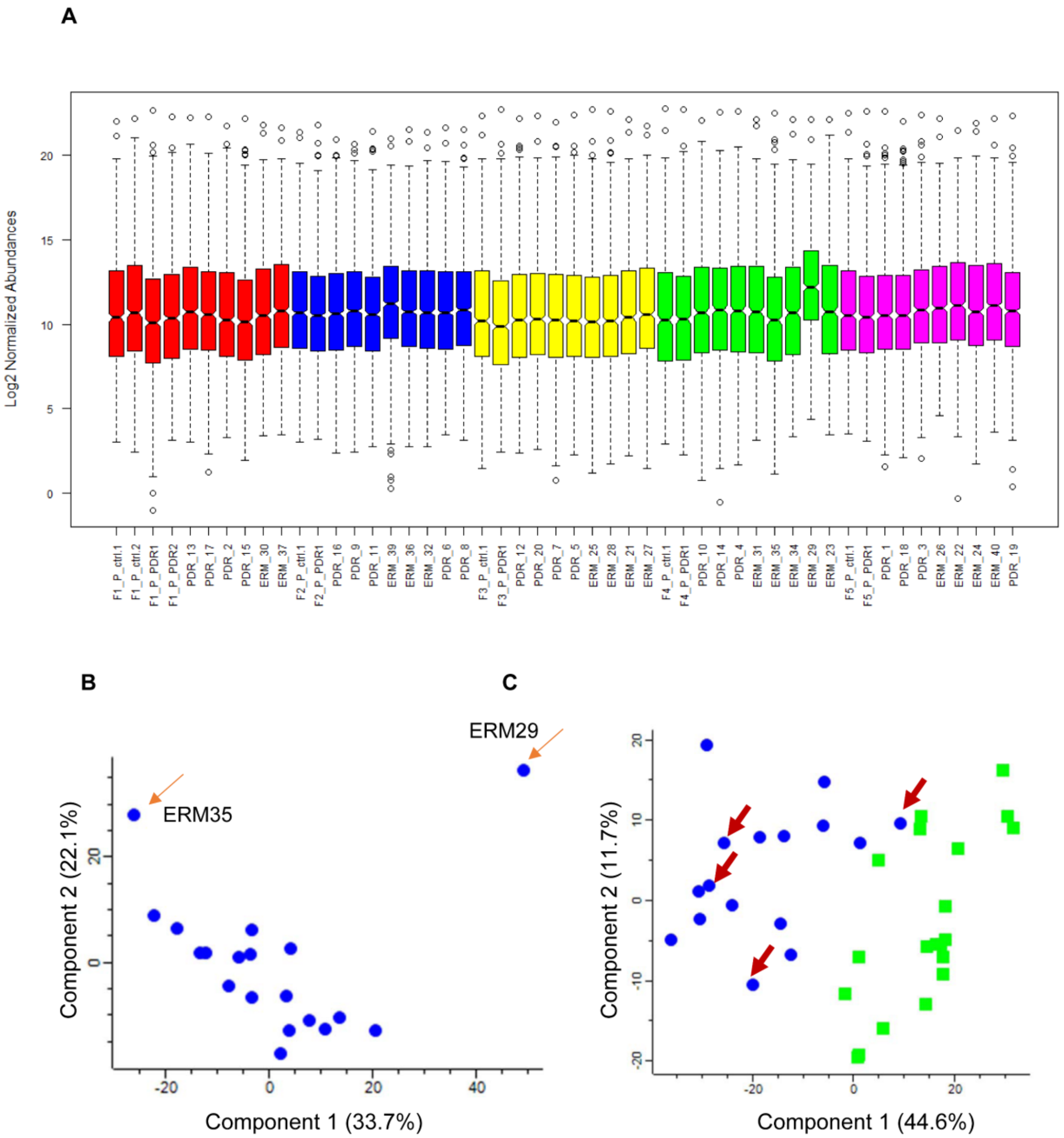

Figure S1. Proteome profiling of PDR vitreous. (A) Boxplot showing ratio distribution of all channels from TMT-based proteome profiling of all PDR and ERM samples. (B) Two outliers (orange arrows) from ERM samples identified through principal component analysis (PCA). (C) PCA plot showing the distribution of the four ERM samples with diabetes (red arrows) among the other ERM and PDR samples. Blue dots denote all ERM samples and green squares denote PDR samples.

A

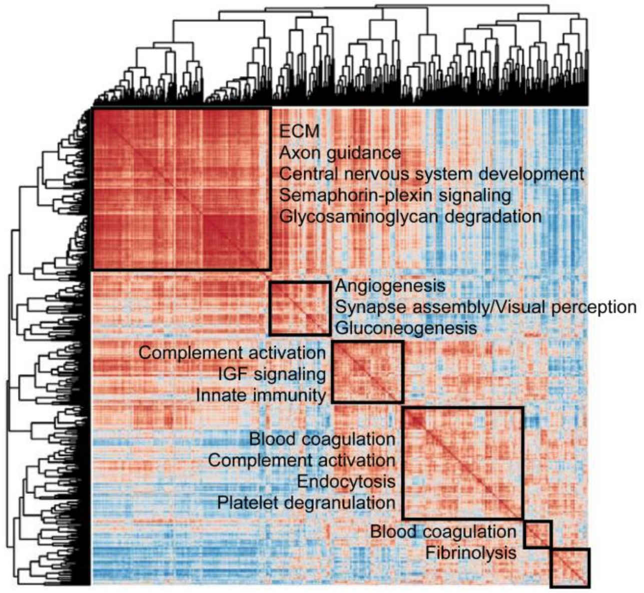

B

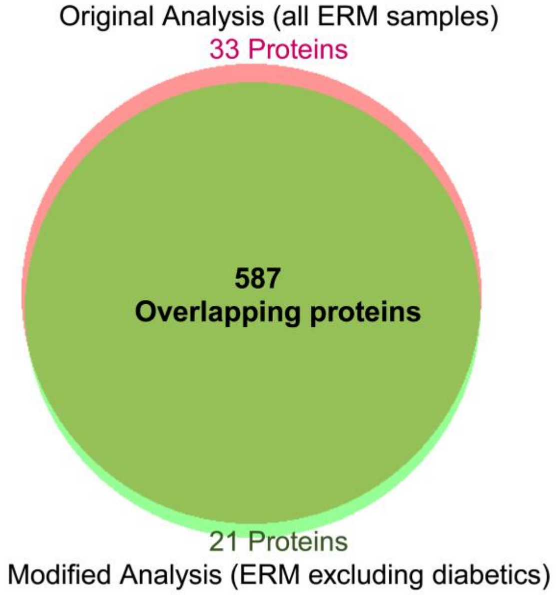

Figure S2. Proteome landscape of vitreous. (A) Correlation map of control ERM group. Global correlation map of all proteins across the ERM samples by assessing the Pearson correlation coefficients of all protein combinations. Highly correlated protein clusters are highlighted along with their functional annotation terms. Positive correlations are indicated by deep brown and negative correlations are shown as blue. (B) Differential expression analysis comparing overlap of significantly altered proteins between PDR and all ERM samples (including diabetic ERM), and PDR and non-diabetic ERM samples (excluding four diabetic ERM samples).

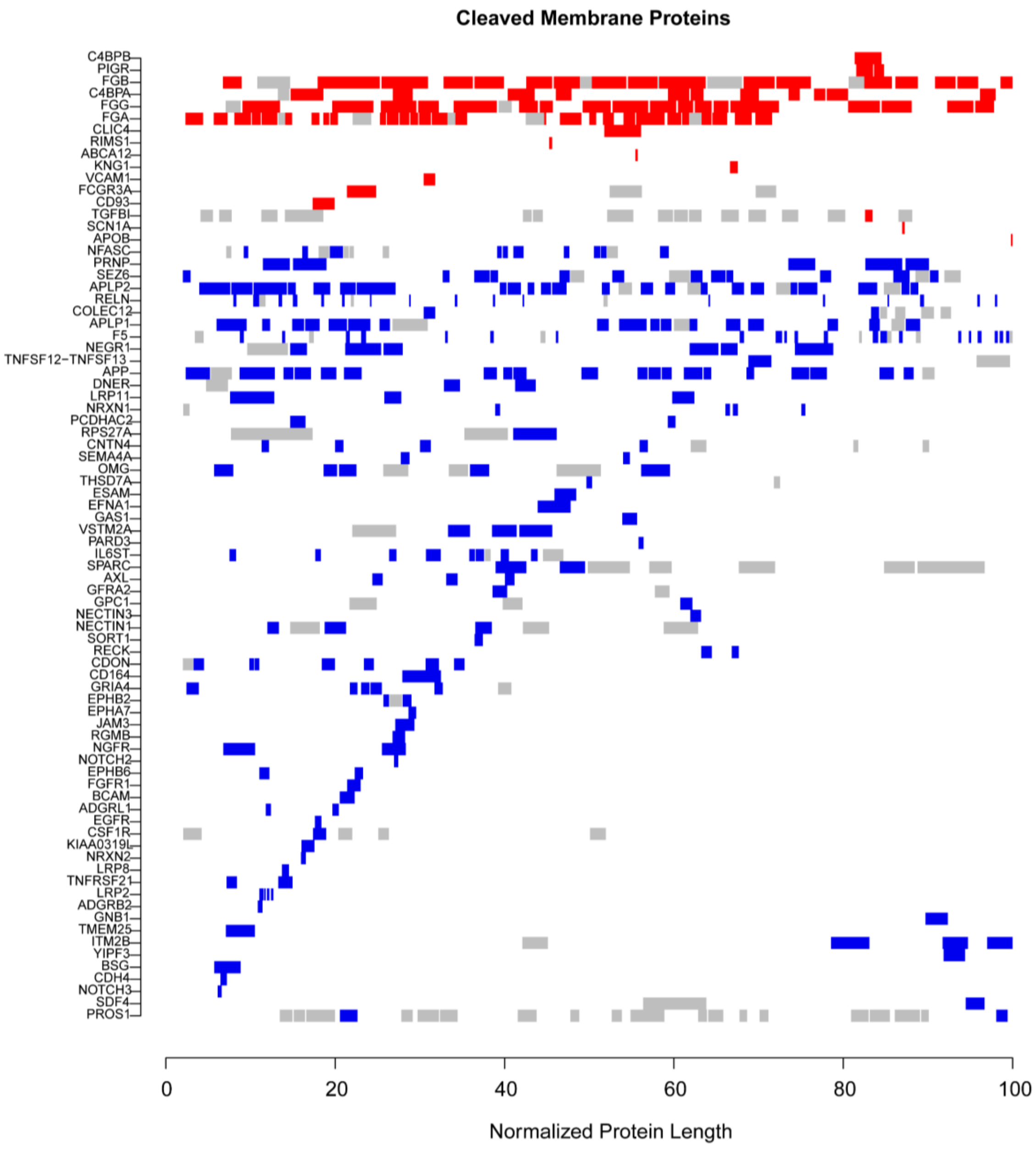

Figure S3. Impaired ectodomain processing of membrane-bound receptor proteins. Proteins displaying differential proteolytic cleavage across the PDR and the control ERM groups are shown. Peptides shown in blue and red indicate reduced or increased abundance by 1.5-fold in the PDR group, and those in grey indicate no change in peptide levels between the two groups. The protein lengths are normalized to a scale of 100 for visualization.

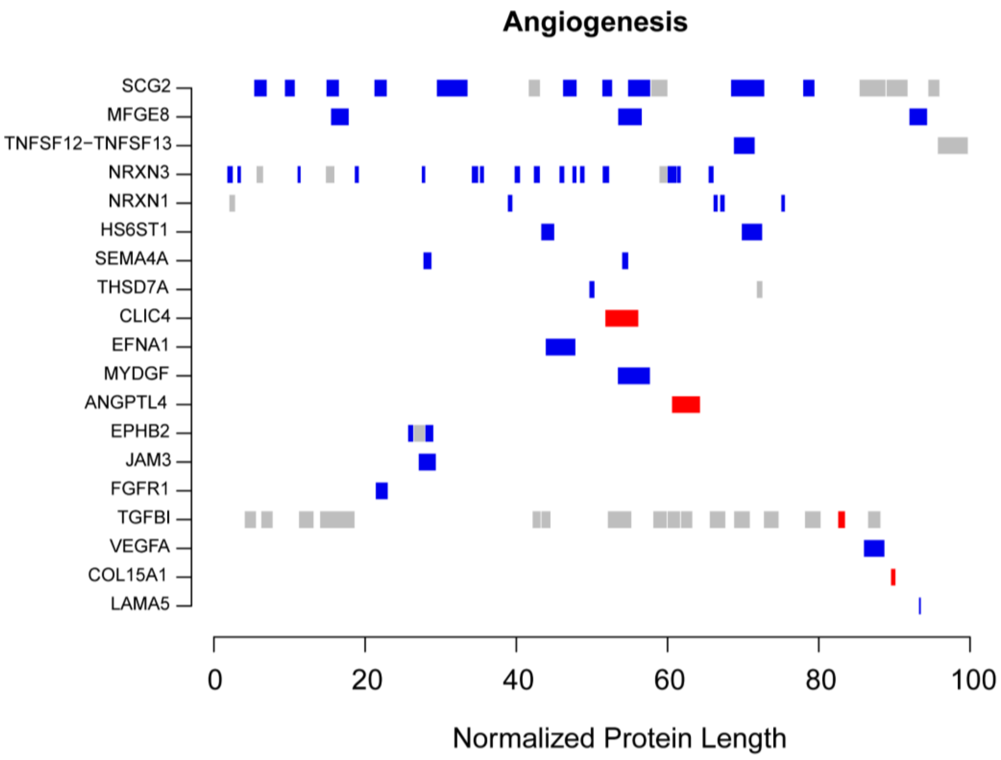

Figure S4. Impaired ectodomain processing of angiogenic proteins. Proteins displaying differential proteolytic cleavage across the PDR and the control ERM groups are shown. Peptides shown in blue and red indicate reduced or increased abundance by 1.5-fold in the PDR group, and those in grey indicate no change in peptide levels between the two groups. The protein lengths are normalized to a scale of 100 for visualization.

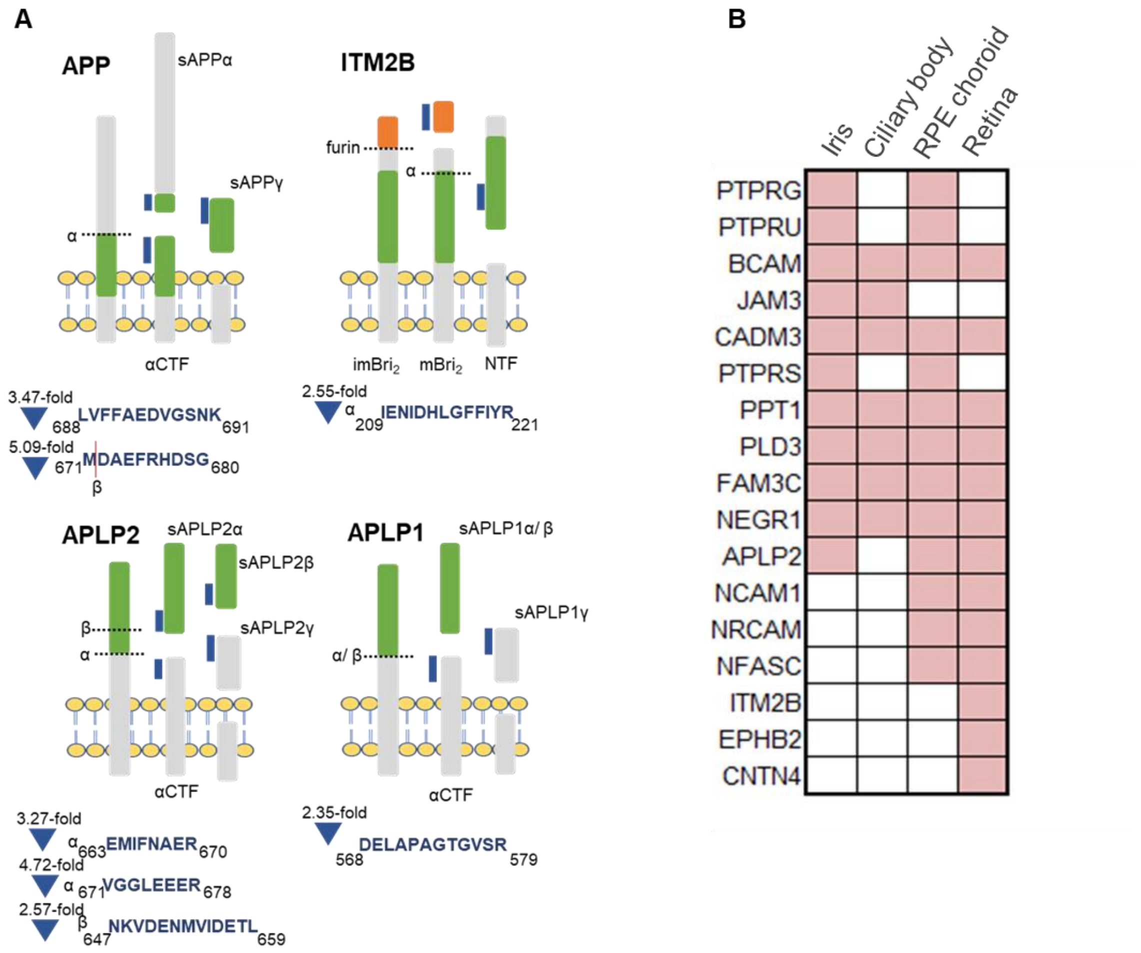

Figure S5. Impaired shedding of ADAM10 substrates in PDR. (A) Substrate processing by specific sheddases and site-specific cleaved peptides detected by MS are highlighted. Only peptides processed by α- and β-secretase are indicated with corresponding fold changes. Inverted blue triangle indicates reduced expression of peptide by the indicated fold. (B) Mapping of ADAM10 substrates to possible localizations in different eye tissues are shown as a heatmap. Pink indicates known expression of indicated protein substrate in the corresponding ocular tissue.

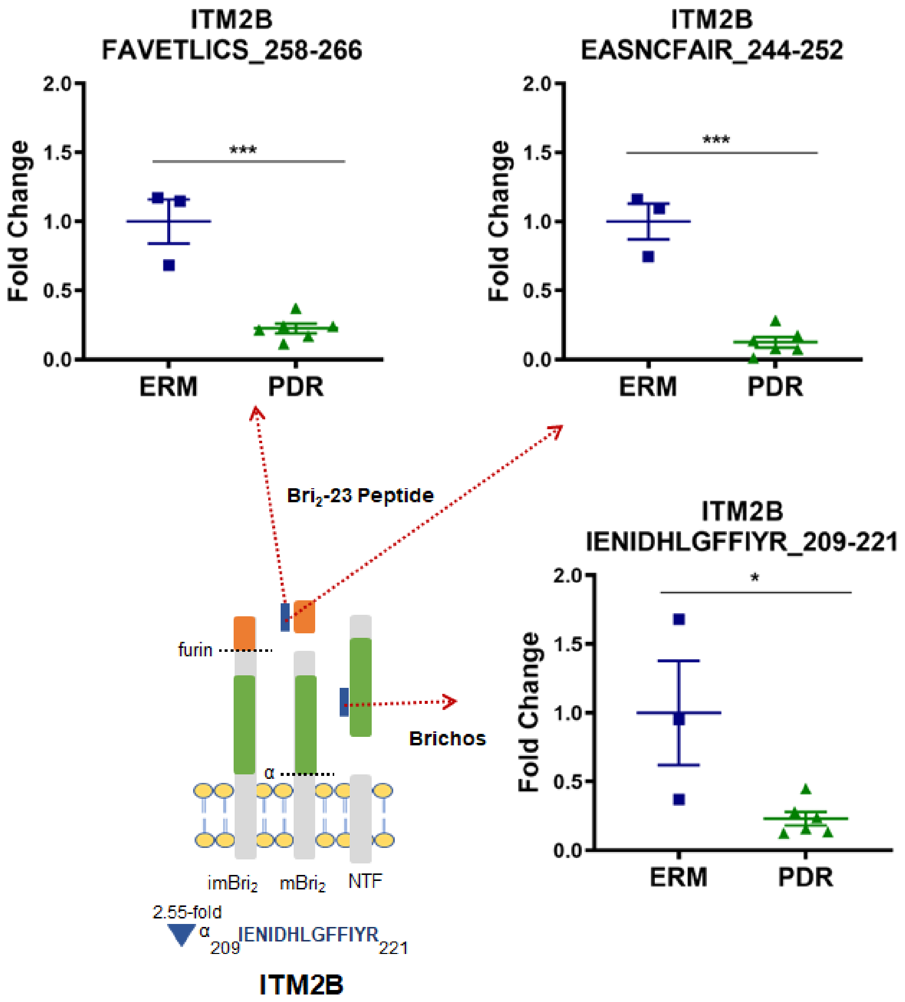

Figure S6. Validation of impaired ITM2B substrate cleavage. Targeted proteomics assay to monitor specific peptide pre-processed by furin and subsequently by ADAM10 in ITM2B in control ERM and PDR groups. Fold change reduction from ADAM10-cleaved substrate region is shown. Data are presented as mean ± s.e.m. Statistical analysis was determined by unpaired, two-tailed Mann Whitney test; *$p < 0.05$, ** $p < 0.01$, and *** $p < 0.001$.

Supplementary Tables:

Table S1: Clinical demographics data.

Table S2. Vitreous proteome profiling of PDR and ERM groups.

Table S3. Significantly altered proteins in PDR vs ERM vitreous.

Table S4. Protein-protein interactions from altered vitreous proteins.

Table S5. Overall peptides quantified from vitreous proteome.

Table S6. Putative proteolytically cleaved protein substrates from altered vitreous proteome.

All supplementary tables are provided as excel (.xlsx) files.
